# The role of 1,4-palladium migration in C(sp^3^)–H functionalization

**DOI:** 10.1039/d5ra05835j

**Published:** 2025-10-21

**Authors:** Mohammad Sadegh Karimtabar, Yasin Mohammadkhani Kalooei, Fatemeh Doraghi, Mohammad Mahdavi, Parviz Rashidi Ranjbar

**Affiliations:** a School of Chemistry, College of Science, University of Tehran Tehran Iran; b Endocrinology and Metabolism Research Center, Endocrinology and Metabolism Clinical Sciences Institute, Tehran University of Medical Sciences Tehran Iran momahdavi@sina.tums.ac.ir

## Abstract

The design of methods for C(sp^3^)–H functionalization of organic compounds is significantly vital for researchers, as they can facilitate the synthesis of various compounds essential for future applications, including natural products and pharmaceuticals. This advancement provides vital tools for creating diverse organic compounds necessary for innovation in drug design and development. Palladium is considered the most effective and commonly employed transition metal in the construction of organic compounds. In recent years, it has demonstrated the ability to catalyze a variety of C–H activation approaches. Palladium-catalyzed C–H activation methods provide notable benefits compared to those facilitated by other metals, as the formed C–palladium bond can take part in numerous follow-up transformations, leading to a broad variety of products. This versatility maximizes the utility and diversity of C–H functionalization processes. One strategy that aids in the functionalization of C(sp^3^)–H bonds is the 1,4-palladium migration. Furthermore, the most frequently observed palladium migration is the 1,4-migration. In this type of migration, the palladium moves from one carbon atom to another carbon atom that is separated by two intervening atoms. This process tends to be favored because it can lead to the formation of a relatively strain-free five-membered palladacycle and is also promoted by the palladium's close proximity to the C–H bond. This type of migration plays a crucial role in the functionalization of C(sp^3^)–H bonds, particularly in molecules equipped with traceless directing groups. In this review, the focus is on the impact of 1,4-palladium migration in C(sp^3^)–H functionalization and how this process selectively activates specific hydrogen atoms within organic compounds.

## Abstract 2

C(sp^3^)–H activation is a major area in organic synthesis. Palladium's versatility makes it essential for C–H activation. We explored 1,4-palladium migration as a common metal migration pathway in C(sp^3^)–H functionalization.

## Graphical abstract

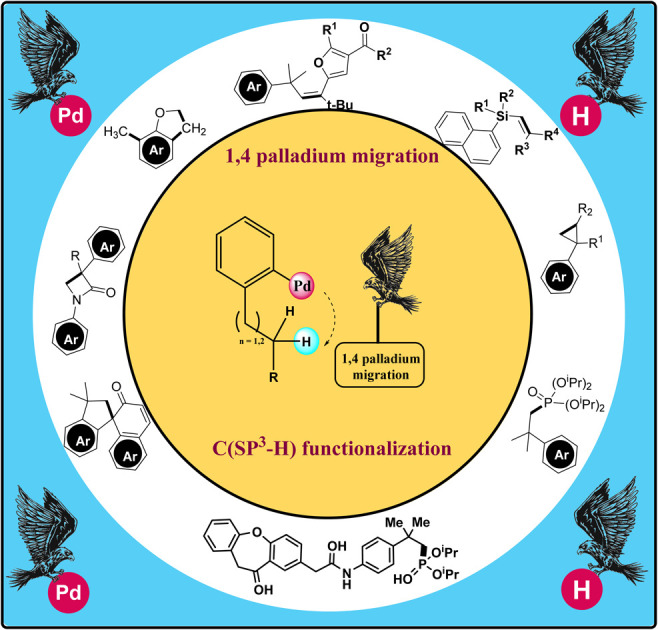

## 1. Introduction

C–H activation is a notably challenging but essential technique in organic chemistry. C–H bonds are strong, non-polar, and typically unreactive. These characteristics make C–H activation a difficult process. However, when successful, it can significantly influence organic synthesis.^1–5^ Incorporating the C–H bond into the catalog of “functional groups” modifies the retrosynthetic strategy and greatly changes the approach of synthesis in organic chemistry by expanding the available synthetic routes and improving total efficiency in the reaction. C–H functionalization allows us to transform C–H bonds into various functional groups using cost-effective methods.^6,7^ Given that numerous organic compounds are ultimately derived from fossil fuels that consist of C–H and C–C bonds, effective C–H activation has the potential to revolutionize the industrial production of organic compounds and improve the efficiency of utilizing these nonrenewable resources. Numerous transition metals have been found to facilitate C–H activation.

Palladium, commonly recognized as the most broadly used transition metal in organic synthesis, has demonstrated a strong ability to drive a wide range of C–H activation processes, particularly the coordination-assisted activation of C(sp^3^)–H bonds.^8^ Palladium-catalyzed C–H activation processes offer a significant advantage over those catalyzed by many other metals, primarily because the C–palladium intermediate formed during activation can be readily engaged in a wide range of follow-up transformations. This versatility enhances both the efficiency and the product diversity of C–H functionalization, enabling straightforward progress from C–H activation to diversified bond-forming. While metals such as Rh, Ru, Co, Cu, and Ni can also promote C–H activation in certain contexts, documented examples with these metals are comparatively fewer, and their reaction manifolds are often less generalizable than those established for palladium. Consequently, palladium-catalyzed C–H activation remains the most broadly explored and practically applied pathway in this area, with the C–palladium bond serving as a versatile handle for subsequent cross-coupling, functionalization, or rearrangement steps.^9^

**Fig. 1:**
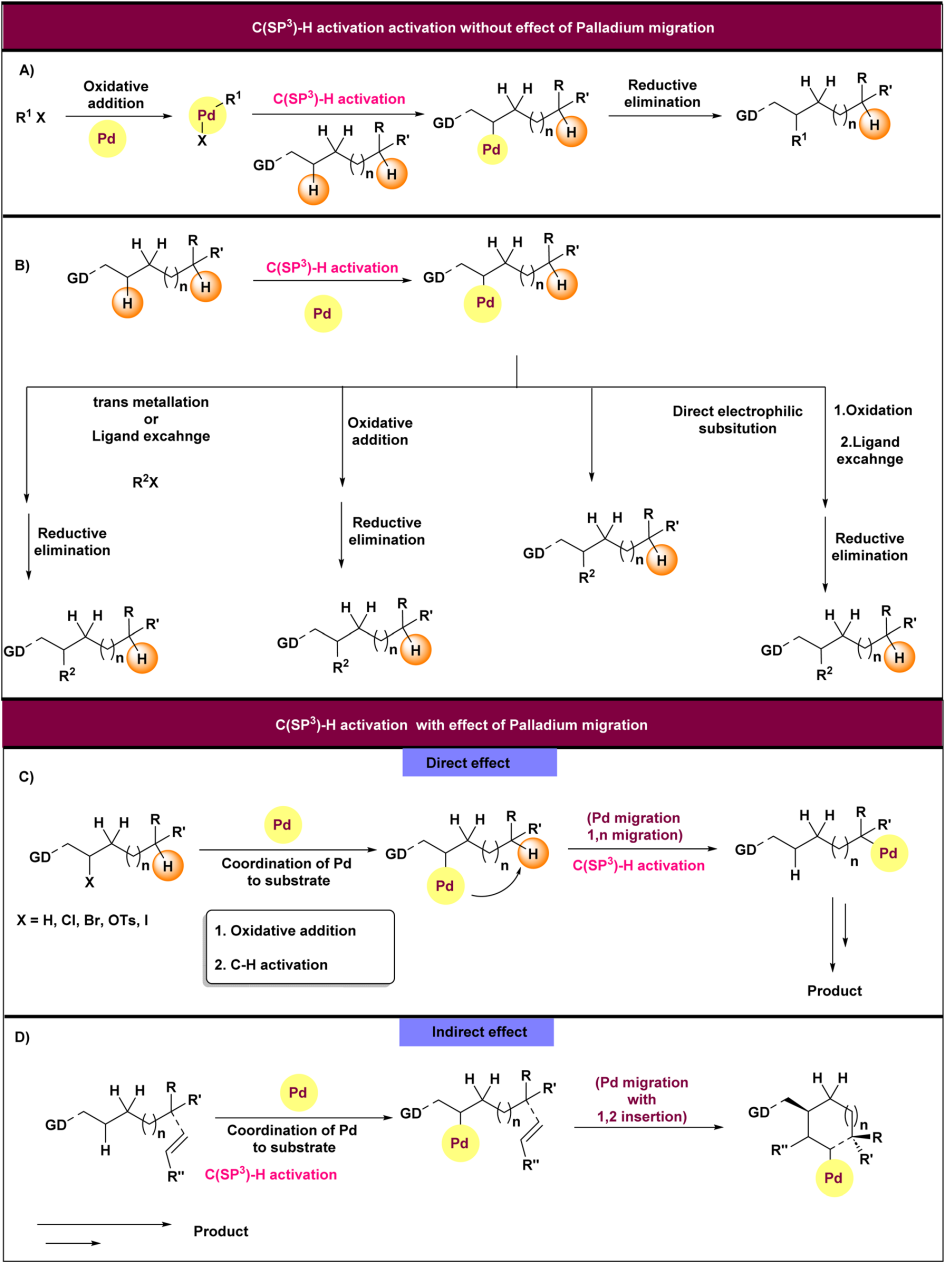
C–H activation strategies with and without palladium migration. (a) C(sp^3^)–H activation without palladium migration and *via* oxidative addition of palladium. (b) C(sp^3^)–H activation without palladium migration and oxidative addition. (c) C(sp^3^)–H activation *via* direct effect of palladium migration. (d) C(sp^3^)–H activation *via* indirect effect of palladium migration.

C(sp^3^)–H functionalization using palladium catalysis provides opportunities to simplify the construction of complex molecules.^10–14^ C(sp^3^)–H activation poses a considerable challenge in synthetic organic chemistry because of the low acidity of these bonds and the relatively weak nature of the metal-alkyl bond. Additionally, the challenge increases when it comes to distinguishing between different C–H bonds in complex substrates. The functionalization of the C(sp^3^)–H bond takes place through two scenarios involving palladium(ii)/palladium (0), palladium(ii)/palladium(ii), and palladium(ii)/palladium(iv) catalytic cycles. In the first route, C(sp^3^)–H activation took place without palladium migration (Fig. 1A and B).^8^ We classify the reactions in this group into two distinct types based on their initial step. The first step may involve the coordination of palladium with the directing group while simultaneously activating the C(sp^3^)–H bond, or it may involve the oxidative addition of palladium to a halide in the substrate. In the second route, the activation involved either direct or indirect effects of palladium migration (Fig. 1C and D). A notable aspect of palladium-catalyzed C–H activation in the first route is that palladium typically targets C–H bonds that are near a directing group, such as an amide, carboxylic acid, ketone, and amine group.^15–31^ While this directing effect provides excellent selectivity in C–H activation processes, it greatly restricts their synthetic applicability to functionalization of near C–H bonds. Consequently, researchers have been exploring methods to use palladium for the activation and functionalization of “remote” C–H bonds that are not close to a directing group. In this context, palladium migration through space occurs significantly more efficiently on C–H bonds that are situated at a greater distance from the directing group.^32–34^ Following the migration of palladium, additional reactions take place that eliminate the palladium element from the structure and functionalize the activated C–H bond. This functionalization can be achieved through a range of appropriate synthetic approaches, such as ligand exchange, elimination, nucleophilic displacement of Palladium(ii), and transmetalation. However, up to now we have only observed elimination and nucleophilic displacement and ligand exchange following 1,4-palladium migration, with other methods not detected.^32,35–39^ for example Buchwald and coworkers in 2005 observed a ligand exchange after 1,4-palladium migration and larock and coworkers in 2008 also showed a nucleophilic displacement of palladium(ii) and elimination after 1,4-palladium migration.^40,41^ It has been observed that palladium migration does not consistently facilitate the functionalization of remote C–H bonds and sometimes was used for C–H bond near to directing group.^33,42^ There are instances where palladium migration (1,2-palladium migration) plays a role in the mechanisms for the functionalization of C(sp^3^)–H bonds adjacent to directing groups.^42–45^ The 1,4-palladium migration is a frequently occurring process in C(sp^3^)–H functionalization, involving the formation of a five-membered palladacycle intermediate. This mechanism facilitates the activation of more distant hydrogen atoms that are otherwise challenging to target using traditional methods. We consider examples of C(sp^3^)–H functionalization that were completed with the assistance of 1,4-palladium migration. This type of migration was usually done with a traceless directing group that could be Br, I, or OTf. Generally, the process begins with palladium acting as a catalyst to insert into the C(sp^2^)–TDG bond through oxidative addition, forming a C(sp^2^)–palladium–TDG intermediate that positions palladium near the targeted C(sp^3^)–H bond. Next, palladium activates the C(sp^3^)–H bond, resulting in the replacement of hydrogen with palladium and forming a C,C-palladacycle. This intermediate feature a C(sp^2^)–palladium–C(sp^3^) linkage, which is commonly observed in many palladium-catalyzed C(sp^3^)–H functionalization reactions involving TDGs. The C(sp^2^) in the palladacycle can be protonated, facilitating the 1,*n*-palladium migration and breaking its bond with the aryl ring. This migration is influenced by the distance between the C(sp^2^)–TDG and the C(sp^3^)–H bond, with most studies reporting a 1,4-palladium shift as a critical step. Afterwards, a functionalization agent (–FG) reacts with the palladium attached to the C(sp^3^) atom, and reductive elimination then yields the final product featuring a C(sp^3^)–FG bond. This review indicates that 1,4-palladium migration has been developed for forming C–C, C–N, C–P, and C–O bonds, with some instances occurring alongside cyclization and others not. A key limitation for applying 1,4-palladium migration to C(sp^3^)–H functionalization is its reliance on traceless directing groups within the molecules. Although the review demonstrates that 1,4-palladium migration is highly effective for C(sp^3^)–H functionalization and is illustrated by many examples, further development is needed to extend this approach to other directing groups, such as transient directing groups or directing groups that are nonremovable or removable. This method has also been employed in the synthesis and modification of certain drugs, including the synthesis of (±)-lemborexant and the modification of ioexpac and repaglinide.^46,47^

## 2. Historic of palladium migration on C(sp^3^)–H functionalization

**Fig. 2:**
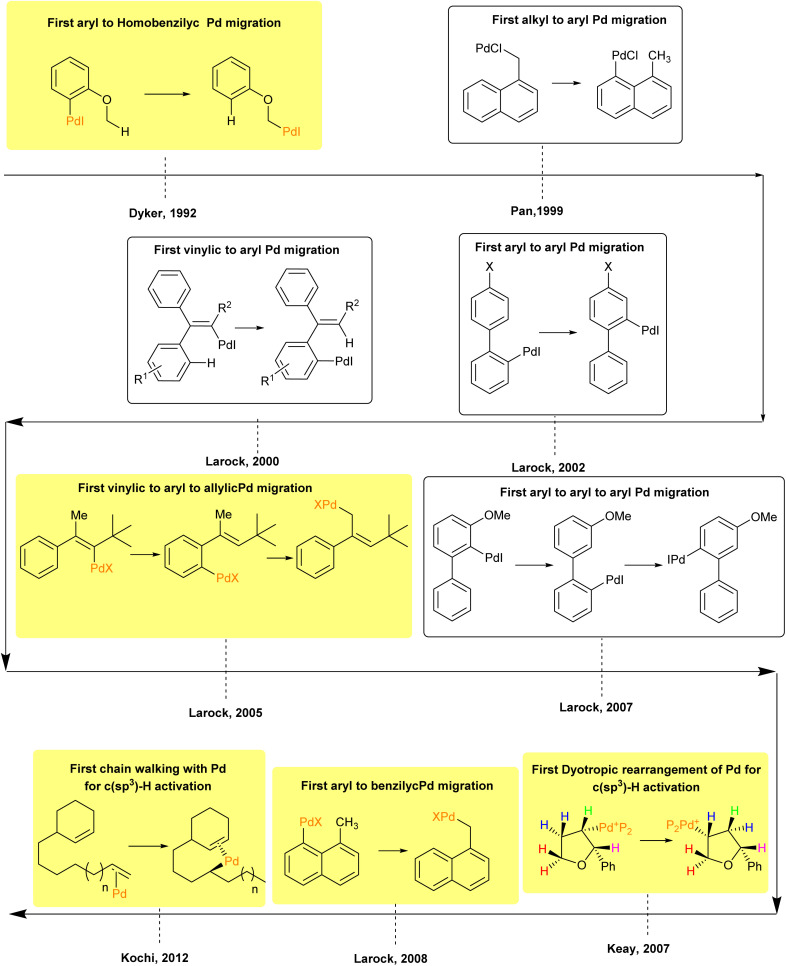
Timeline of the representative discovery and development of palladium migration in C–H activation.

Palladium migration was first observed by Dyker in 1992, where it was noted that palladium migrates from an aryl position to a homo benzylic position.^48^ After that, the researcher explored additional types of Palladium migration that could enable the functionalization of remote C(sp^3^)–H and C(sp^2^)–H bonds in the substrate (Fig. 2). Fig. 2 highlights the migration of palladium for C(sp^3^)–H bond functionalization in the yellow box and C(sp^2^)–H bond in the white box. This review exclusively focuses on the Responsibility of 1,4-palladium migration in facilitating the C(sp^3^)–H functionalization of an organic compound. It is noteworthy that the first sequential migration of palladium was reported by Dyker in 2005 and 2007.^49,50^ Furthermore, the initial observations of chain walking and dyotropic rearrangement of palladium for C(sp^3^)–H functionalization were seen by Kochi and Keay in 2012 and 2007, respectively.^51,52^ In 2005, Larock developed a method for C(sp^3^)–H functionalization involving two 1,4-palladium migrations, transitioning from aryl to vinylic and then to allylic positions for the first time.^49^ Later, in 2008, he published the first report of a 1,4-palladium migration from an aryl to a benzylic position.^9,40^

## 3. Application of 1,4-palladium migration on C(sp^3^)–H functionalization

**Fig. 3:**
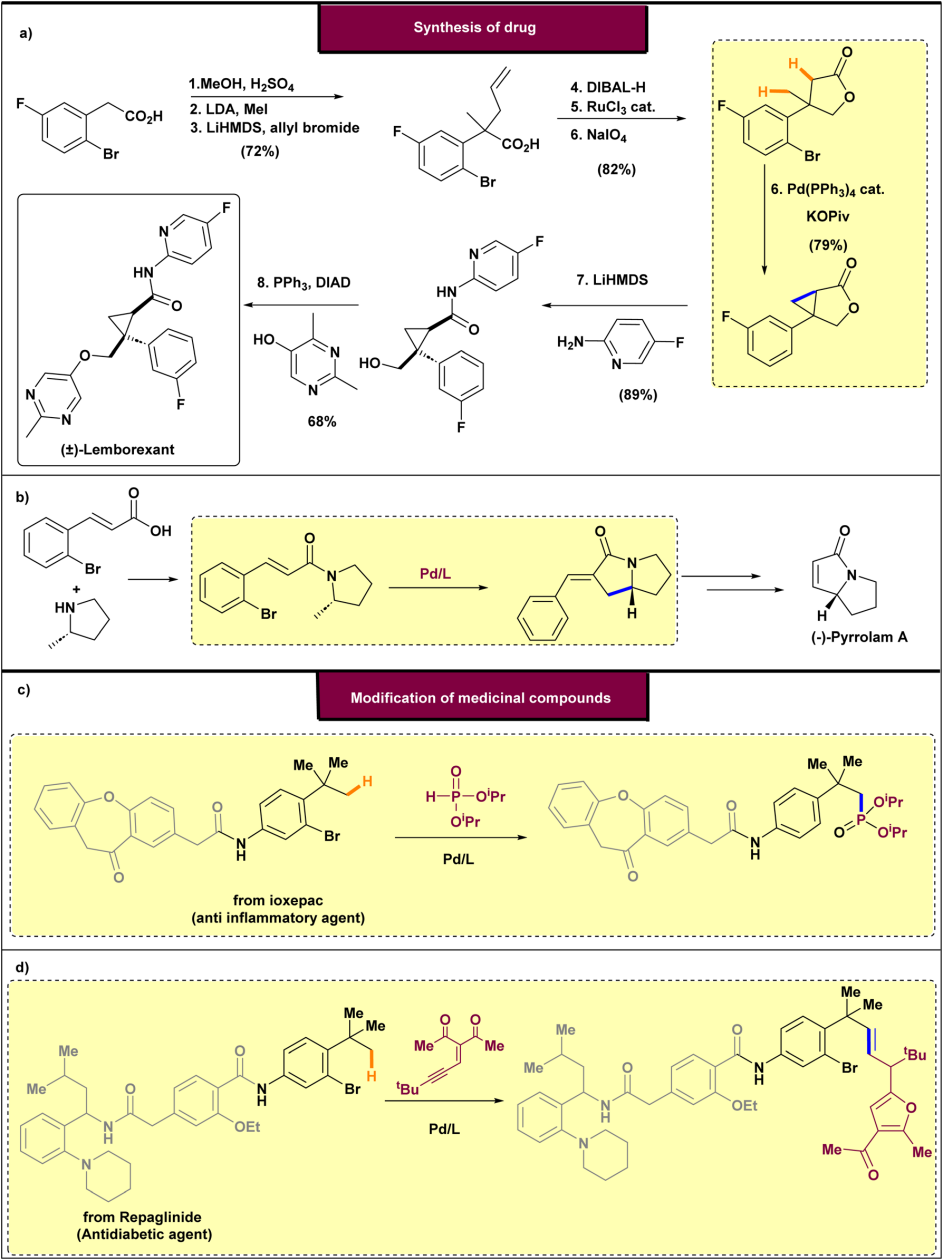
Application of 1,4-palladium migration on C(sp^3^)–H functionalization. (a) Synthesis of (±)-lemborexant. (b) Synthesis (−)-pyrrolam A. (c) Modification of ioxepac (anti-inflammatory agent). (d) Modification of repaglinide (antidiabetic agent).

The 1,4-palladium migration is a vital and widely studied process in the field of organometallic chemistry, particularly in C(sp^3^)–H functionalization. This migration involves the movement of palladium from an initial carbon position to a remote carbon atom that is separated by two intervening atoms. It is an important mechanism because it enables activation of distant C–H bonds that are typically inert and difficult to functionalize directly. The process is often stabilized by the formation of a five-membered palladacycle, which is strain-free and thermodynamically favorable. Numerous examples have demonstrated that palladium can migrate from vinylic, aryl, or alkyl positions—including benzylic and homobenzylic sites—to various functional groups such as aryl, alkyl, acyl, and imidoyl groups. This versatility allows a broad range of transformations, either within a single substrate or through multi-component coupling reactions. Since the pioneering work of Dyker, research efforts have predominantly focused on halogen-based traceless directing groups (TDGs) for C(sp^3^)–H activation. These studies generally follow a similar mechanistic pathway, involving five well-defined steps. Initially, palladium inserts into the C(sp^2^)–TDG bond *via* oxidative addition, positioning it close to the target C(sp^3^)–H bond. Next, palladium activates the C(sp^3^)–H bond, forming a palladacycle with a characteristic C(sp^2^)–palladium–C(sp^3^) linkage (Fig. 3C). Protonation of this intermediate facilitates the key 1,4-palladium migration, which breaks the original bond with the aryl ring. The efficiency of this migration depends on the spatial distance between the C(sp^2^)–TDG and the C(sp^3^)–H site, with many studies emphasizing the significance of the 1,4-shift as a critical step. Subsequently, the addition of a functionalization reagent, followed by reductive elimination, yields the final product with a new C(sp^3^)–FG bond. Overall, the 1,4-palladium migration offers a powerful approach to selectively activate and functionalize remote C–H bonds, broadening the scope and utility of palladium catalysis in organic synthesis. This mechanism continues to be a focus of ongoing research, enhancing our understanding of C–H activation processes. It is noteworthy that this effect of palladium migration in C(sp^3^)–H functionalization has been observed in the synthesis of various drugs, such as (±)-lemborexant and (−)-pyrrolam A, as well as in the modification of different medications, including ioxepac and repaglinide (Fig. 3). In these examples, it was observed that palladium migration facilitated the functionalization of remote C–H bonds in complex molecules that would not be achievable without this migration, as the target C–H bond is located far from the directing group. Therefore, observing various examples of the 1,4-palladium migration on C(sp^3^)–H bonds can guide us in the future to build a novel approach for the construction and modification of drugs using more efficient and accessible material approaches.

## 4. 1,4 Palladium migration

It is very difficult to activate aliphatic C(sp^3^)–H bonds because they are exceptionally strong (BDE > 400 kJ mol^−1^) and very resistant to deprotonation (p*K*_a_ > 40).^53^ Additionally, selectively targeting a specific C–H bond among so many similar bonds in a given environment is difficult.^54^ Recent advancements in directed C–H activation have introduced innovative strategies to solve this problem. One strategy that has gained considerable attention is 1,4-palladium migration. These strategies enable functionalization at positions typically inaccessible by traditional methods.^55^ 1,4-palladium migration presents additional challenges compared to heteroatom-directed C–H activation. To begin with, in contrast to the one-step activation process facilitated by a heteroatom, 1,4-palladium migration involves a series of C–H activation and protonation steps to produce byproducts. Additionally, the two palladium species participating in the migration process exhibit similar reactivity. This, combined with the reversible nature of the migration, makes it difficult to prevent side reactions that deplete the original palladium. Third, under certain conditions, reductive elimination of the necessary palladacycle intermediate may take place.^46^ Despite these obstacles, considerable development has been achieved in this research area over the last 20 years. Most subsequent transformations following palladium migration have focused on reactions involving carbon nucleophiles or insertion.^56–58^

### 4.1. C–C coupling by 1,4 palladium migration

**Scheme 1:**
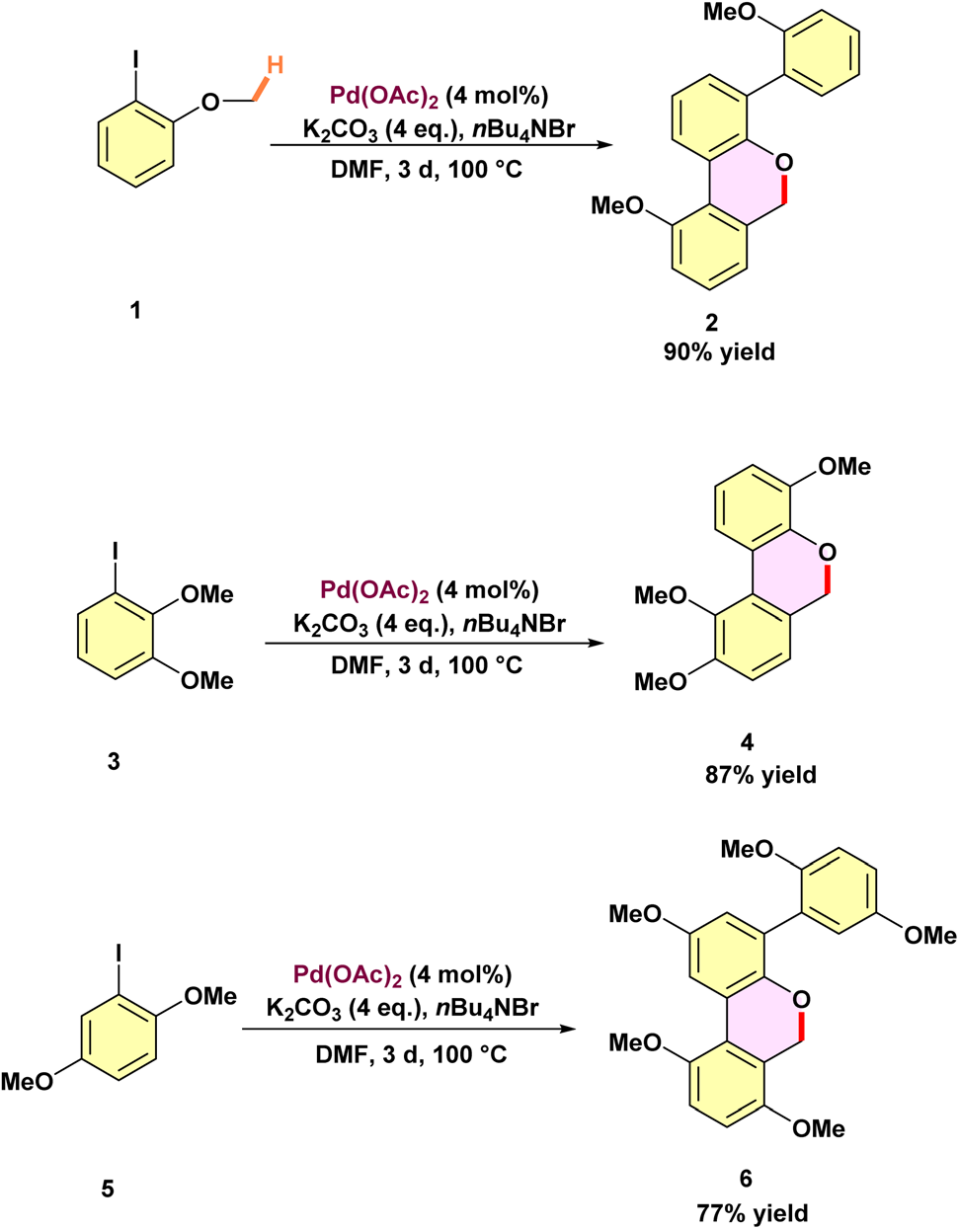
Palladium-catalyzed C–H activation of methoxy groups of substituted 6*H*-dibenzo[*b*,*d*]pyrans.

**Scheme 2:**
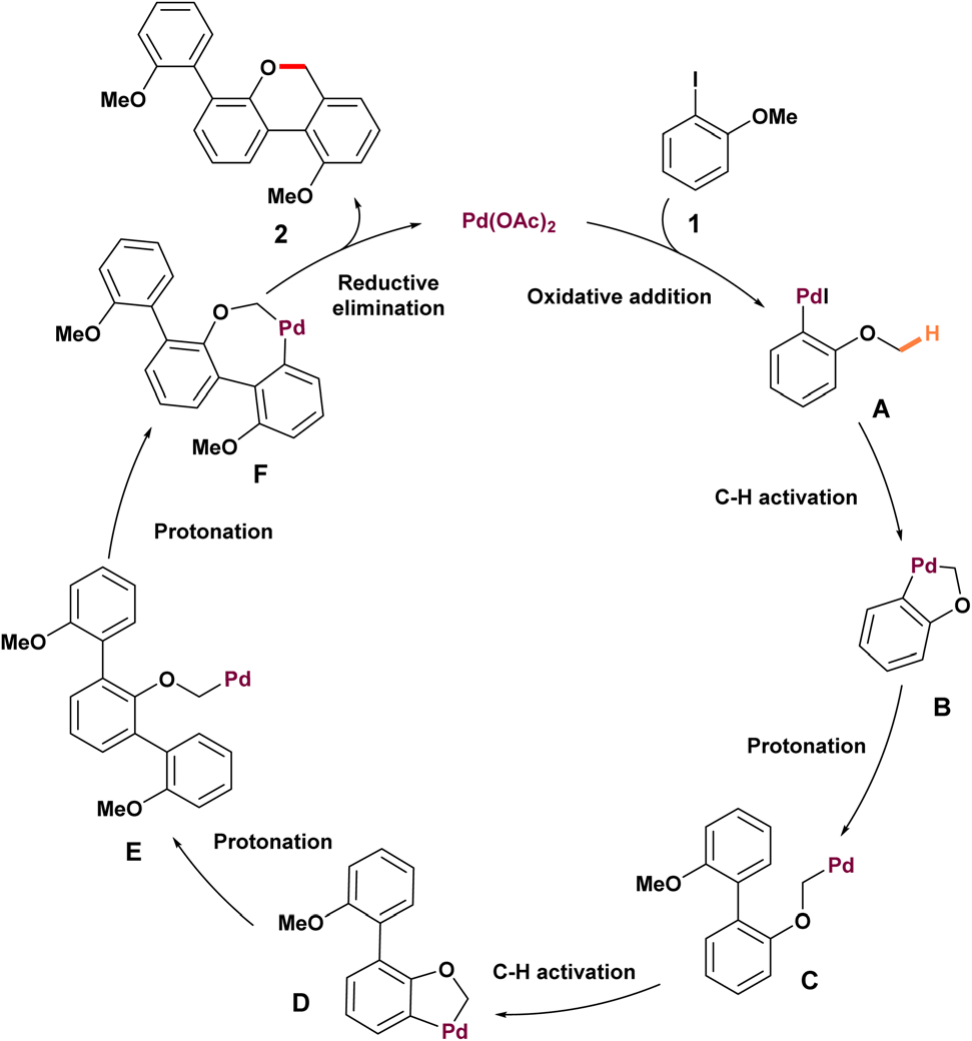
Mechanism of palladium-catalyzed C–H activation of methoxy groups through 1,4-palladium migration.

The concept of palladium migration was first introduced by Dyker in 1992.^48^ His work opened new doors in organic synthesis and laid the foundation for further advancements in catalytic transformations. *Ortho*-iodoanisole 1 undergoes condensation with palladium catalysis to yield a 90% product 2 (Scheme 1). To assess the generality of this annulation and determine the regiochemical outcome, a series of accessible 1-iodo-2,*n*-dimethoxybenzenes were tested. In the case of 1-iodo-2,3-dimethoxybenzene 3, the condensation product 4 was obtained, derived from just two molecules of the precursors. This observation suggests that a key position for the third condensation step is blocked. When 1-iodo-2,5-dimethoxybenzene 5 was subjected to the same reaction, it regioselectively produced 6 as the only isomer, in 77% yield. However, when 1-iodo-2,6-dimethoxybenzene was used, no condensation product was formed, and the starting material was restored in 95% yield. This observation suggests the formation of stabilized palladium complexes, which interrupt the catalytic cycle. The proposed reaction mechanism involves the cyclometallation of aryl palladium(ii) iodide A to form a five-membered ring intermediate B, triggered by intramolecular C–H activation (Scheme 2). Intermediate B undergoes 1,4-palladium migration, leading to the creation of intermediate C, which sets the stage for further progress in catalytic transformations. A subsequent cyclometallation of intermediate C with dehydrohalogenation is expected to produce a five-membered ring D. Repeated addition of compound 1 leads to the symmetric intermediate E, which undergoes cyclometallation to form a heptacyclic compound F, followed by reductive elimination to produce the final product 2.

**Scheme 3:**
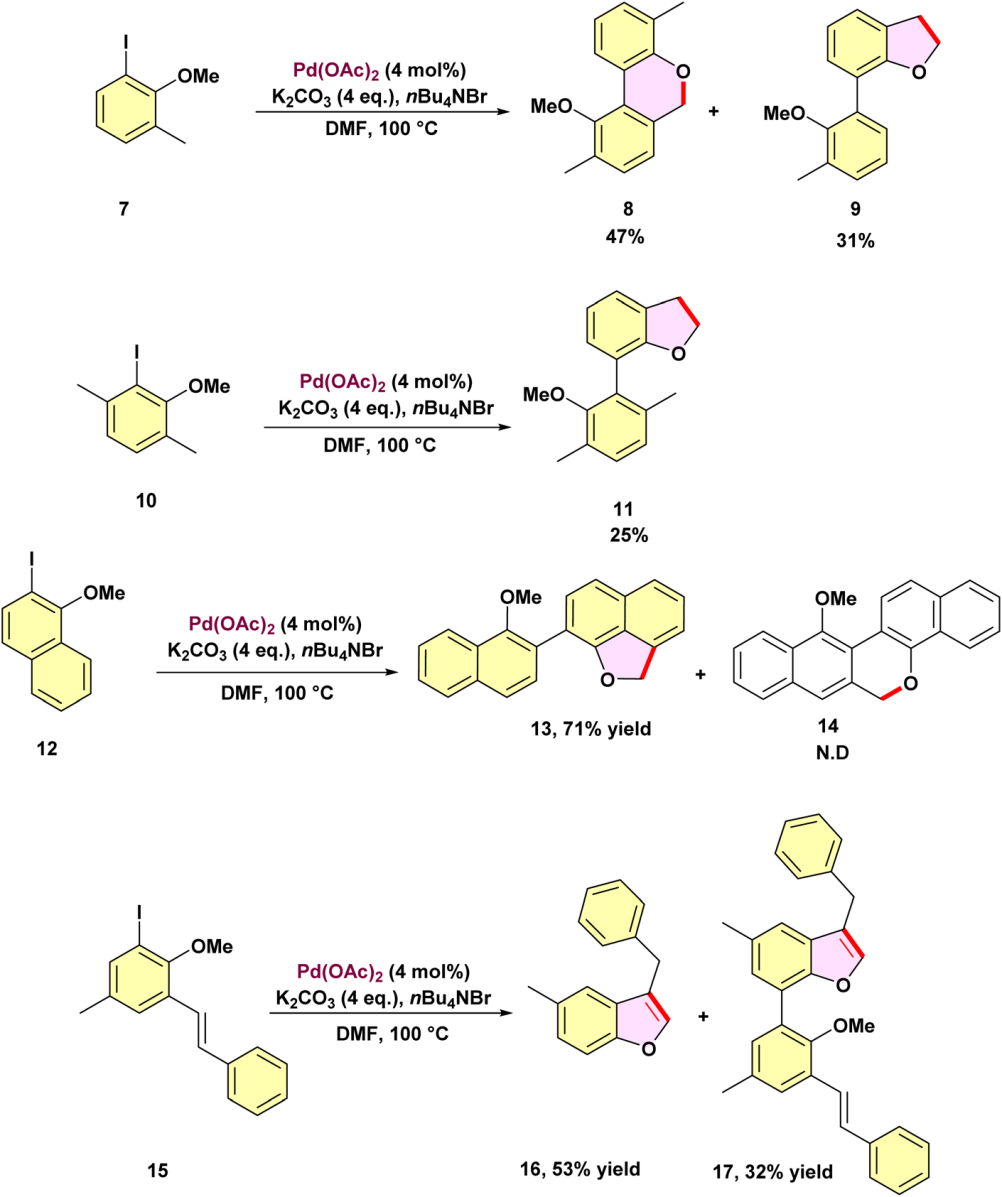
Palladium-catalyzed C–H activation at methoxy groups: regiochemistry of the domino coupling process.

In 1994, he investigated a list of 3-substituted 1-iodo-2-methoxybenzenes homocoupling reactions facilitated by palladium-catalyzed to examine how these substituents influence the regiochemistry of the domino coupling process (Scheme 3).^59^ Similar to the reaction involving the anisole derivative 7 was transformed into a substituted dibenzopyran 8 as the primary product, yielding 47%. Unexpectedly, a side product, 2,3-dihydrobenzofuran 9, was obtained at a yield of 31%. This indicates that the methyl group at the 3-position introduces a new synthetic route, directly contributing to the domino coupling process. For the substituted *ortho*-iodoanisole 10, the presence of an additional methyl group likely inhibits the dibenzo[*h*,*d*]pyran formation by obstructing a key position, and the benzofuran 11 became the dominant product, yielding 25%. When the benzo-annulated precursor 12 was used, the naphthofuran 13 was produced in good yield, with no formation of the dinaphthopyran 14 observed. Similarly, the coupling reaction of the functionalized stilbene 15 did not produce a pyran derivative. Instead, the aromatized benzofuran 16 was isolated as the major product. The spectroscopic data for the byproduct, including both ^1^H,^1^H–COSY and ^1^H,^13^C–COSY spectra, were consistent with compound 17.

**Scheme 4:**
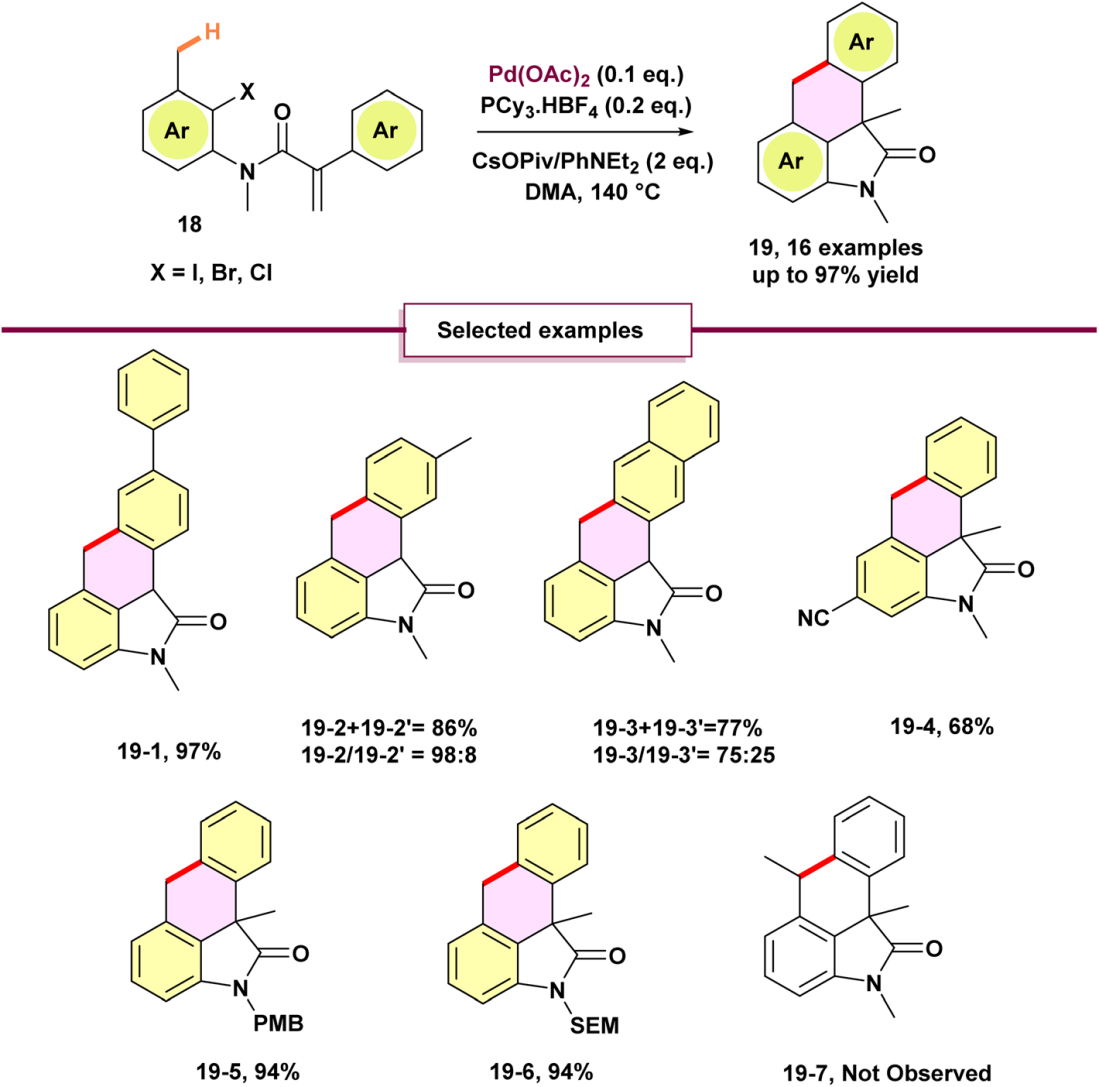
Palladium-catalyzed through-space C(sp^3^)–H and C(sp^2^)–H bond activation by 1,4-palladium migration.

**Scheme 5:**
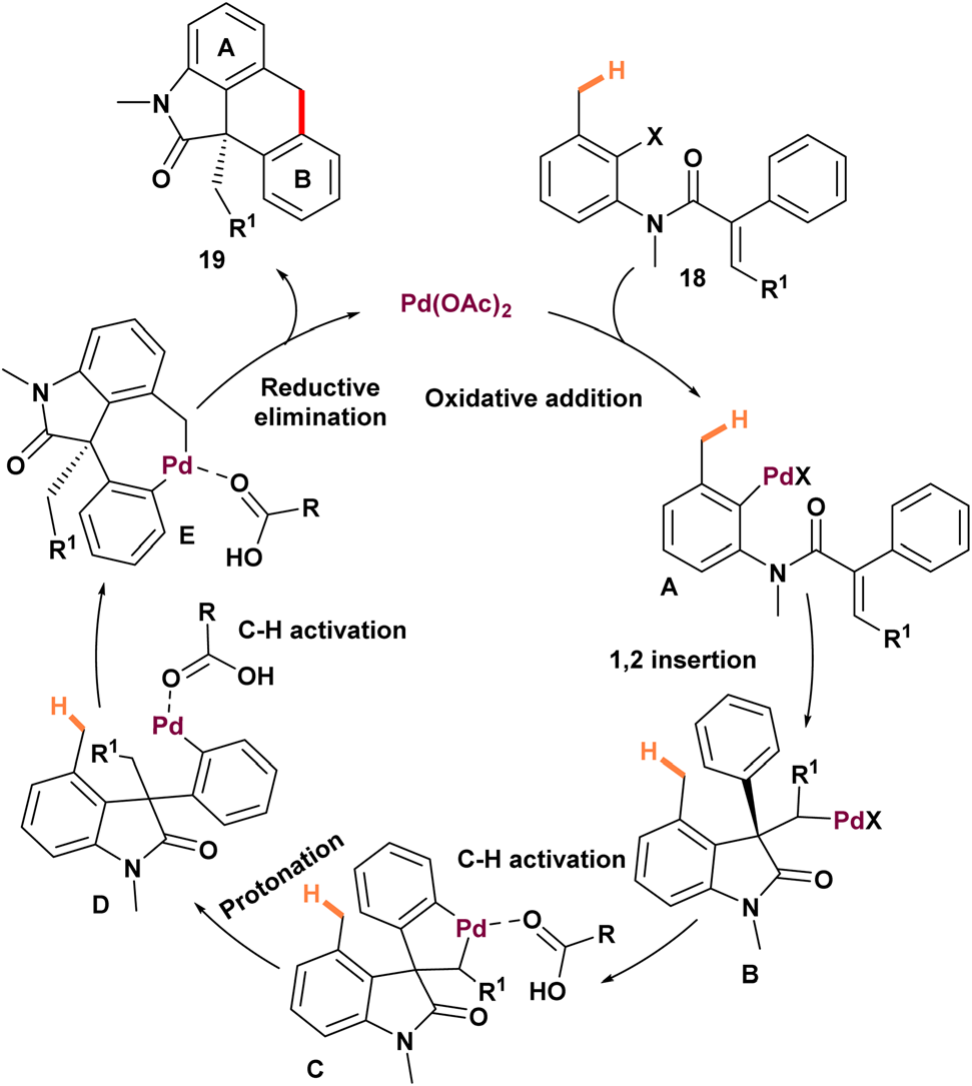
Mechanism of palladium-catalyzed through-space C(sp^3^)–H and C(sp^2^)–H bond activation by 1,4-palladium migration.

Zhu *et al.* reported an efficient novel strategy to synthesize [3,4]-fused oxindoles 19 through 1,4 palladium migration (Scheme 4).^60^ The synthesis of oxindoles presented two primary challenges. First, before 2013, there were limited examples of six-membered ring formation *via* activation of C(sp^3^)–H bonds and subsequent carbon–carbon bond creation. Second, potential side reactions, particularly the formation of benzocyclobutane from a five-membered palladacycle C, competed with the desired palladium migration pathway (Scheme 5). To address these obstacles, extensive optimization of reaction conditions was conducted. Pd(OAc)_2_ combined with PCy_3_·HBF_4_ emerged as the most effective catalyst system, while a 1 : 1 mixture of CsOPiv and *N*,*N*-diethylaniline delivered excellent yields. Employing a tertiary amide proved essential for facilitating the domino reaction and enabling successful double cyclization of *N*-benzyl, *N-para*-methoxybenzyl, *N*-phenyl, and *N*-2-(trimethylsilyl)ethoxymethyl anilides. Though attempts to synthesize 19-7*via* methylene group activation were unsuccessful. A *meta*-methyl group directed cyclization toward the less sterically hindered position, yielding 19-2 with high regioselectivity. Similarly, β-naphthyl-substituted substrates predominantly formed 19-3 as the major product. Oxidative addition of substrate 18 to palladium (0) initiates intramolecular carbopalladation, generating an alkyl palladium(ii) intermediate A (Scheme 5). These compounds activate the adjacent aromatic C(sp^2^)–H bond, forming a five-membered palladacycle B. A formal proton transfer facilitates 1,4-palladium migration from the alkyl to the aryl position. The resulting palladium(ii)-aryl intermediate undergoes C–C bond rotation, activating the C4 methyl group and forming a seven-membered palladacycle D. Subsequent reductive elimination produces the tetracyclic oxindole and regenerates the palladium (0) catalyst.

**Scheme 6:**
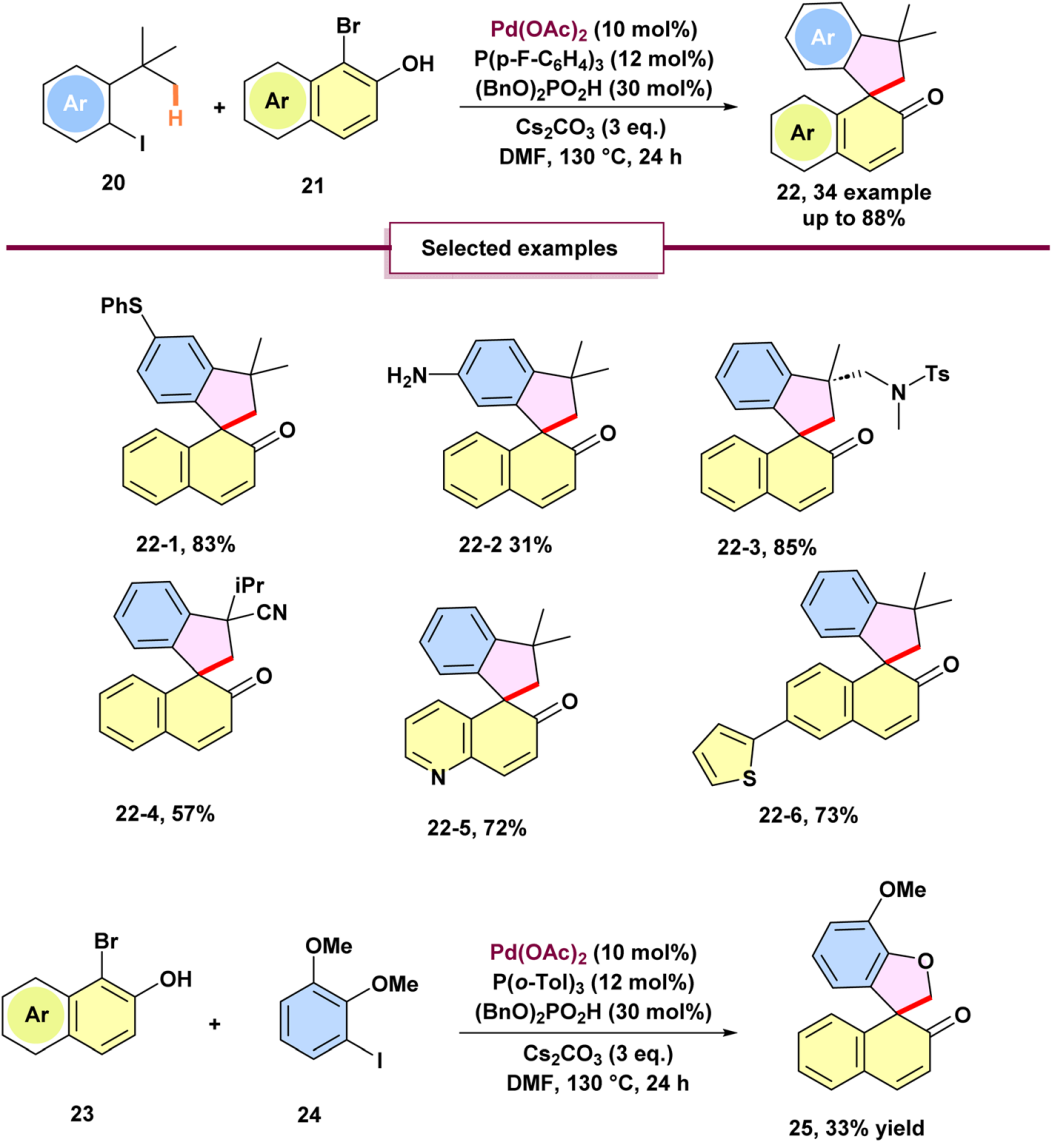
Palladium-catalyzed intermolecular [4 + 1] spiroannulation by C(sp^3^)–H activation and naphthol dearomatization.

Luan *et al.* reported a palladium-catalyzed [4 + 1] spiro annulation *via* a C(sp^3^)–H activation/naphthol dearomatization strategy (Scheme 6).^61^ This transformation exhibited broad substrate scope, tolerating diverse aryl substitutions, including electron-donating, electron-neutral, and electron-withdrawing groups. Notably, the reaction proceeded even in the presence of a strongly coordinating free amine, albeit delivering the spiro annulation product 22-2 in reduced yield. Inspired by Dyker's precedent on palladium(0)-catalyzed homocoupling of 2-iodoanisoles *via* methoxy-directed C–H activation, the authors hypothesized that simple 2-iodoanisoles could serve as four-carbon synthons for the desired [4 + 1] annulation. Compared to previously employed 2-iodo-*tert*-butylbenzenes, 2-iodoanisoles posed additional challenges due to the free rotation of the methoxy group, which is less accessible to the palladium. Interestingly, substrate 24, bearing an *ortho*-substituent adjacent to the methoxy group, enabled a direct bimolecular annulation with 23 to furnish product 25 in 33% yield. However, the efficiency of this transformation was still impacted by the competing homocoupling pathways of the 2-iodoanisole derivatives, which prevented the reaction from achieving a high yield.

**Scheme 7:**
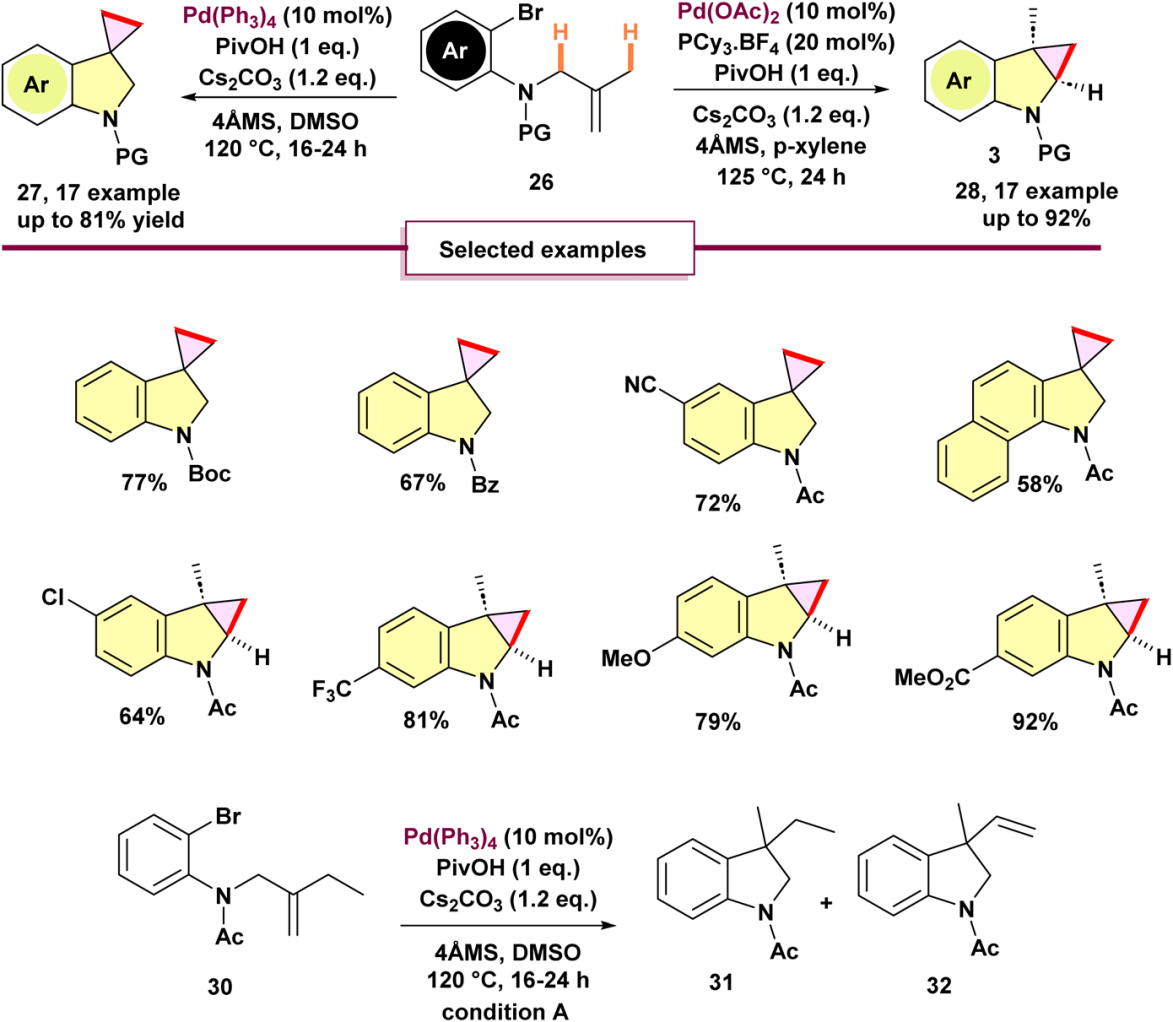
Palladium-catalyzed tandem divergent cyclopropanation *via* solvent-driven regioselective C(sp^3^)–H bond activation.

In 2018, Lee and Baik introduced a unique solvent-driven method through regio-divergent C(sp^3^)–H bond activation for the production of spiro 27 and fused 28 cyclopropanated indolines (Scheme 7).^62^ This approach enabled a one-pot, selective synthesis of two different products: spiro 27 and fused 28 cyclopropanated indolines. 1,4-Palladium migration was not detected during the formation of these two products. However, it was noted that ethylallylated 2-bromoarylamide 30 showed altered under condition A reactivity, resulting in a mixture of vinylated indoline 32 and protonated compound 31 (with a ratio of 116/115 = 10 : 1, as determined by 1H NMR analysis) in 74% yield. This finding indicated that the σ-alkylpalladium(ii) intermediate, formed from 30, passed through 1,4-palladium migration followed by β-H elimination, resulting in 32 as the primary product.

**Scheme 8:**
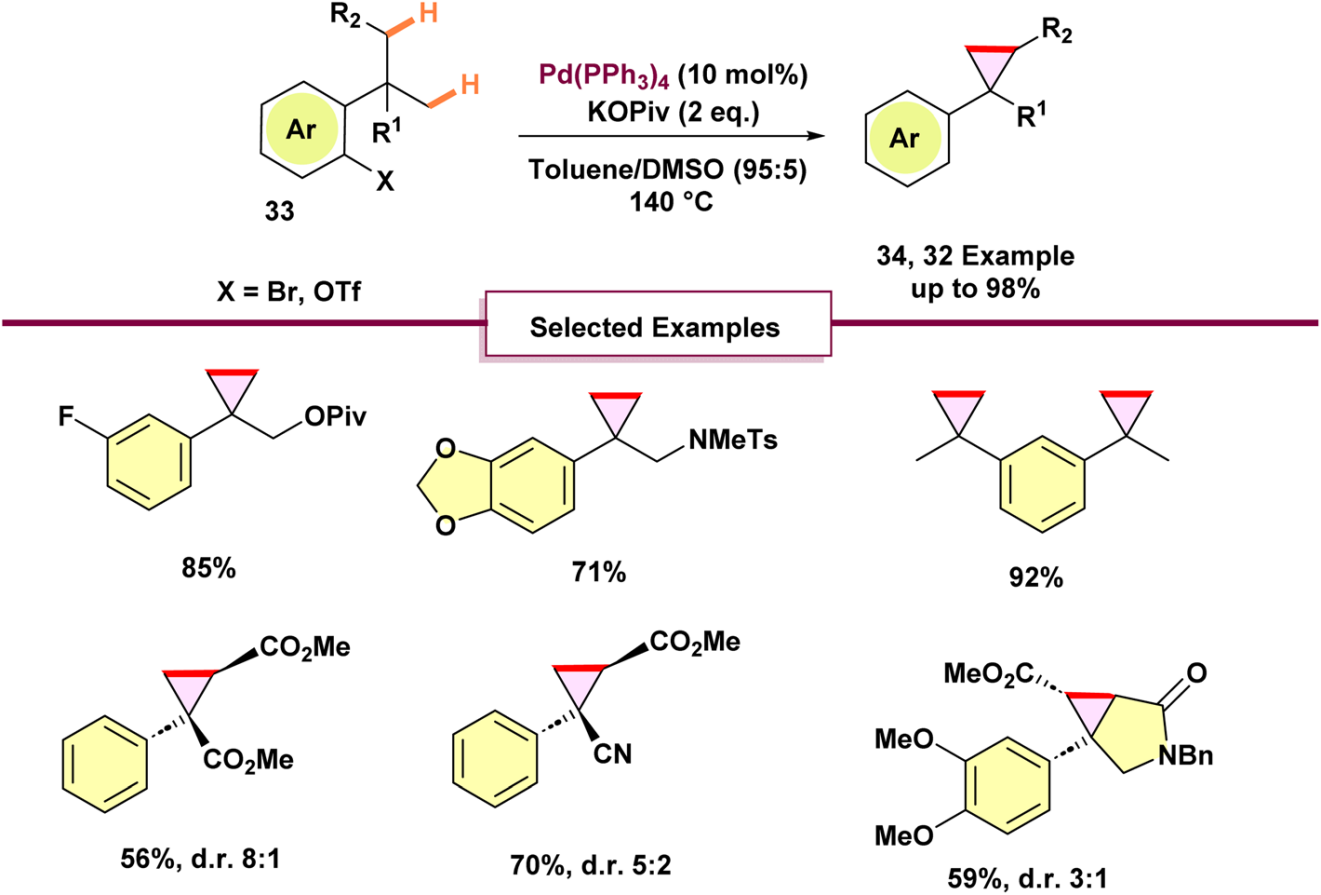
Direct synthesis of cyclopropanes from *gem*-dialkyl groups through double C–H activation.

**Scheme 9:**
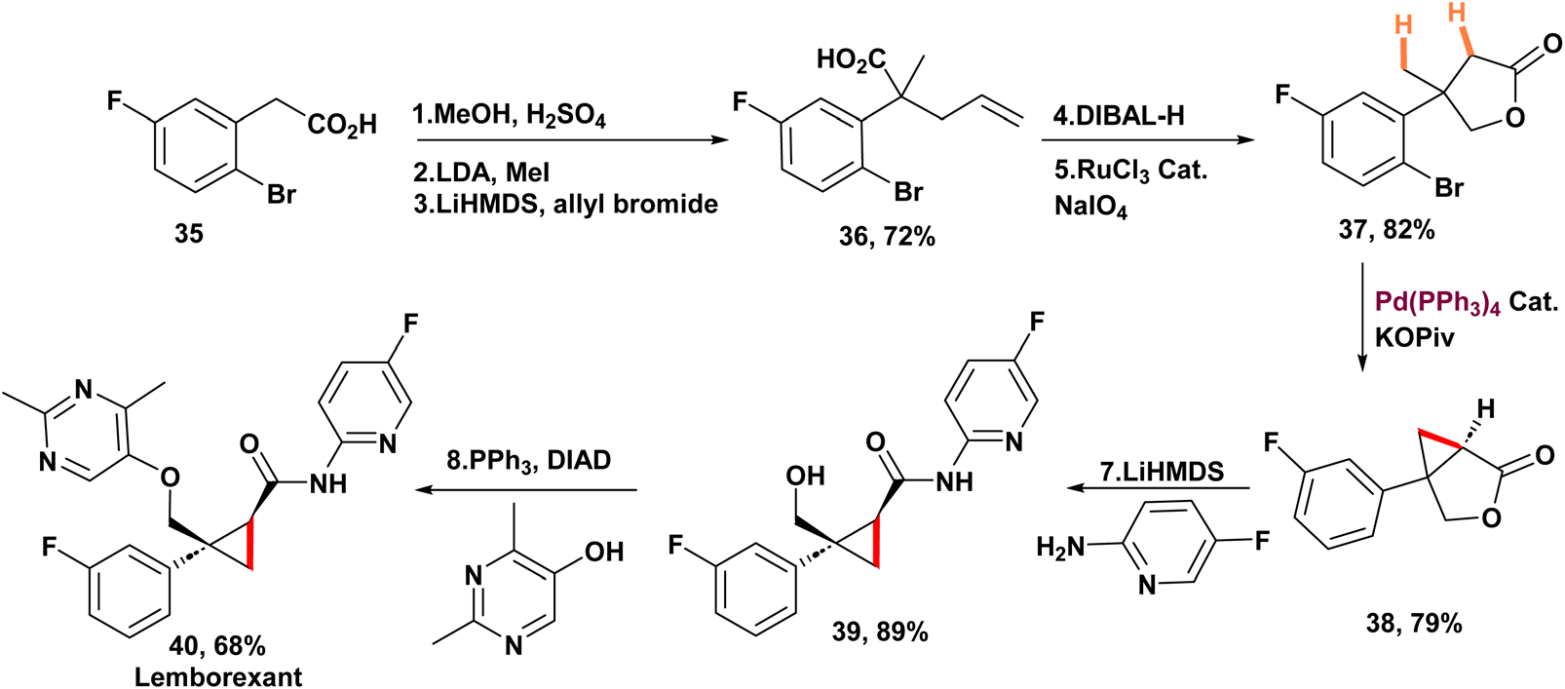
Synthesis of lemborexant *via* 1,4-palladium migration.

In 2021, Baudoin and co-workers disclosed a one-step strategy for the synthesis of cyclopropane scaffold 34*via* the coupling of either two geminal methyl groups or a methyl group with an activated methylene moiety (Scheme 8).^46^ This process exploits the natural behavior of arylpalladium halide species, generated through oxidative addition, to undergo a 1,4-palladium migration, furnishing a σ-alkylpalladium intermediate. Upon optimizing the reaction conditions, the authors demonstrated the synthetic utility of this methodology by applying it to the construction of lemborexant, a recently approved therapeutic agent for insomnia (Scheme 9). Using pivalate as the base is essential to steer the reaction mechanism toward forming the cyclopropane, rather than the other product observed previously.^63,64^ Stoichiometric mechanistic experiments revealed that aryl- and alkyl-palladium pivalates exist in equilibrium through a five-membered palladacycle; with pivalate, a second C(sp^3^)–H activation favors the generation of a four-membered palladacycle intermediate and ultimately the cyclopropane product. The process began with the esterification of aryl bromide 35, which was then followed by a series of reactions involving alkylation and allylation to produce methyl ester 36. The following reduction of the ester and oxidative cleavage of the alkene resulted in the formation of γ-butyrolactone 37. Finally, the key cyclopropanation of 37 under the optimized conditions afforded fused lactone 38 in 79% yield. Direct amidation using aminopyridine and LiHMDS, coming after a Mitsunobu reaction with pyrimidinol, resulted in racemic lemborexant 40 with an overall yield of 28% across eight steps. Notably, a previous report from 2015 had achieved the synthesis of this molecule in only 16% overall yield^65^

**Scheme 10:**
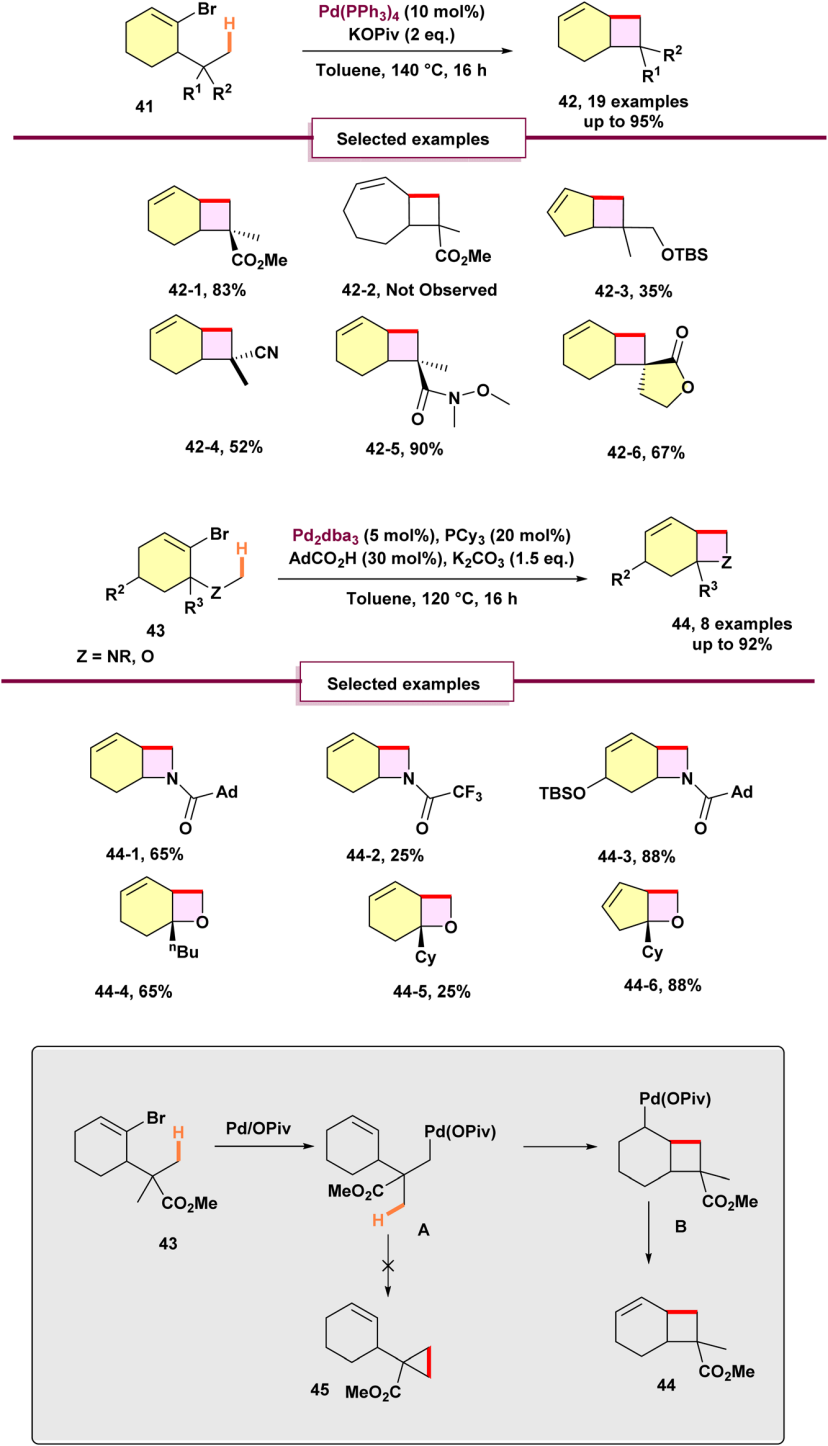
Synthesis of fused 4-membered rings through 1,4-palladium migration.

**Scheme 11:**
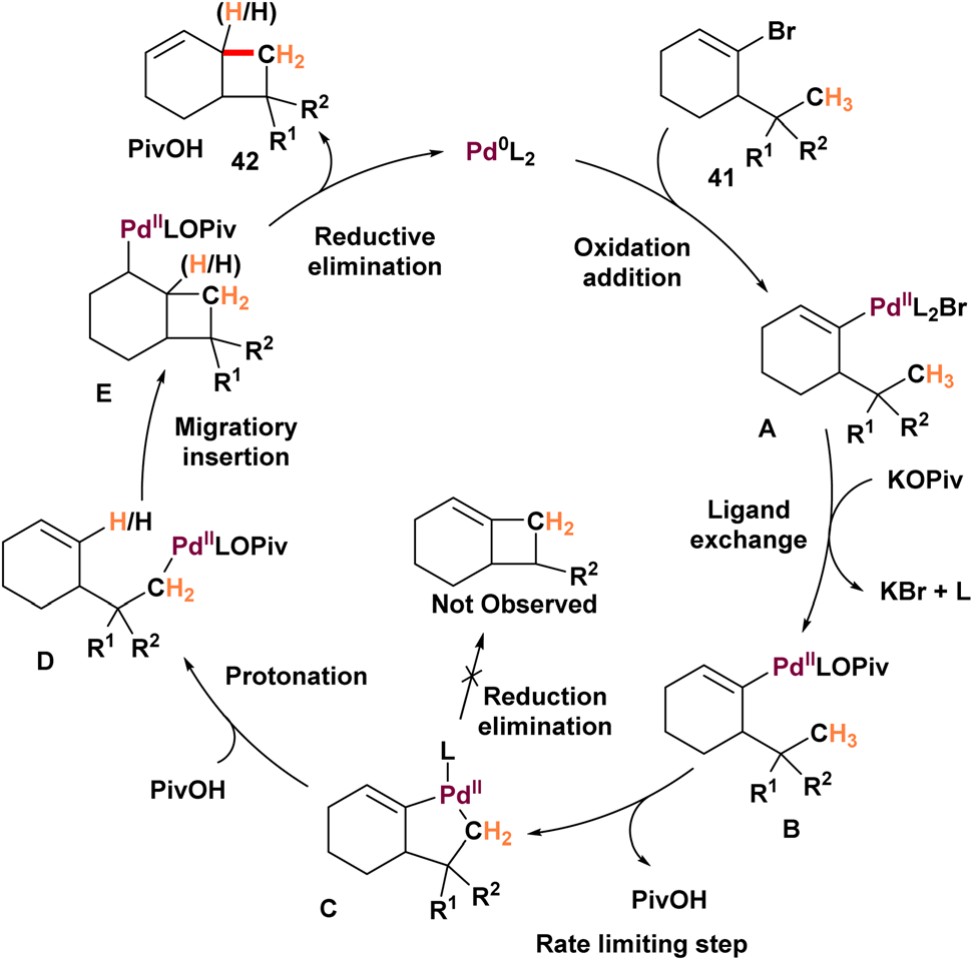
Mechanism of synthesis of fused 4-membered rings through 1,4-palladium migration.

In addition, He conducted research which led to a simple and efficient strategy for the synthesis of fused cyclobutanes 42, azetidines, and oxetanes 44. The method employs cycloalkenyl electrophiles and relies on an unexpected palladium^0^-catalyzed C(sp^3^)–H activation coupled with an alkenyl-to-alkyl 1,4-palladium migration (Scheme 10).^66^ As mentioned earlier, aryl halides bearing geminal alkyl substituents undergo palladium^0^-catalyzed double C(sp^3^)–H activation to afford aryl cyclopropanes. However, the substrate scope of this approach has been largely restricted to aryl-based systems. To overcome this limitation, the authors explored cycloalkenyl electrophiles 41 as alternative coupling partners. They proposed that cycloalkenyl bromide 69 could also undergo an alkenyl-to-alkyl 1,4-Palladium migration, analogous to its aryl counterparts, to generate the σ-alkylpalladium intermediate A. In principle, intermediate A could engage in a second C(sp^3^)–H activation to yield cyclopropane 44. However, this pathway would require the formation of a highly strained four-membered palladacycle, which is energetically disfavored. Instead, the migratory insertion of the alkene into the palladium–C bond of intermediate A is kinetically favored. This process leads to the formation of fused cyclobutane 45 through a β-hydride elimination sequence. This approach ultimately facilitates the synthesis of used cyclobutanes, azetidines, and oxetanes. After the optimization process, the scope of the derivative has been examined. Substrates tolerating gem-dimethyl groups along with an ester, nitrile, Weinreb amide, or protected primary alcohol on the quaternary carbon showed good performance, yielding the corresponding cyclobutane products in moderate to high yields. This method served to synthesize the more complex spirobicyclo[4.2.0]-octen 42-6. The cycloheptenyl substrate did not result in the expected fused cyclobutane 42-2. Using the same method for cyclobutanes, the possibility of synthesizing fused azetidines using cycloalkenyl electrophiles with an *N*-methyl substituent was explored. They explored the possibility of synthesizing fused azetidines by using cycloalkenyl electrophiles that have an *N*-methyl group, similar to their work with cyclobutanes. They designed a precursor 43, which included a 1-adamantylamide group. This design had two main goals: (1) to increase steric hindrance around the nitrogen atom, and (2) to suppress unwanted reactions involving the protecting group on the nitrogen. However, when they replaced this protecting group with trifluoroacetyl, the yield dropped significantly 44-2, demonstrating the influence of having a congested protecting group on the nitrogen atom. For oxetanes 44-4, 44-5, they successfully applied the azetidination conditions to produce the desired products. Interestingly, using a five-membered ring precursor led to higher outcomes, resulting in the formation of oxabicyclo[3.2.0]heptene 44-6 with an impressive yield of 88%. Based on the data, Cycloalkenyl bromide interacts with the active palladium^0^ L_2_ catalyst to form complex A, which undergoes rapid ligand exchange to produce σ-alkenylpalladium pivalate B (Scheme 11). Kinetic data suggest that complex B is likely the catalyst resting state, with the pivalate group coordinating to facilitate the following C–H activation step. C(sp^3^)–H activation in the presence of base then generates palladacycle C. Due to its high energy barrier, reductive elimination from C to form the strained cyclohexene-fused cyclobutane E was unfavorable. Instead, the protonation of C by pivalic acid at the C(sp^2^) carbon, forming σ-alkylpalladium species D, proceeds as the kinetically preferred pathway, completing the two-step 1,4-palladium migration. Subsequently, a *syn*-stereospecific migratory insertion of the olefin in D into the palladium–C bond yields complex E. This complex passes through *syn*-β-H elimination to produce the fused cyclobutane product, regenerating the active catalyst and forming PivOH. Mechanistic studies indicate that the C–H activation step resulting in the formation of intermediate B is both irreversible and the rate-limiting step.

**Scheme 12:**
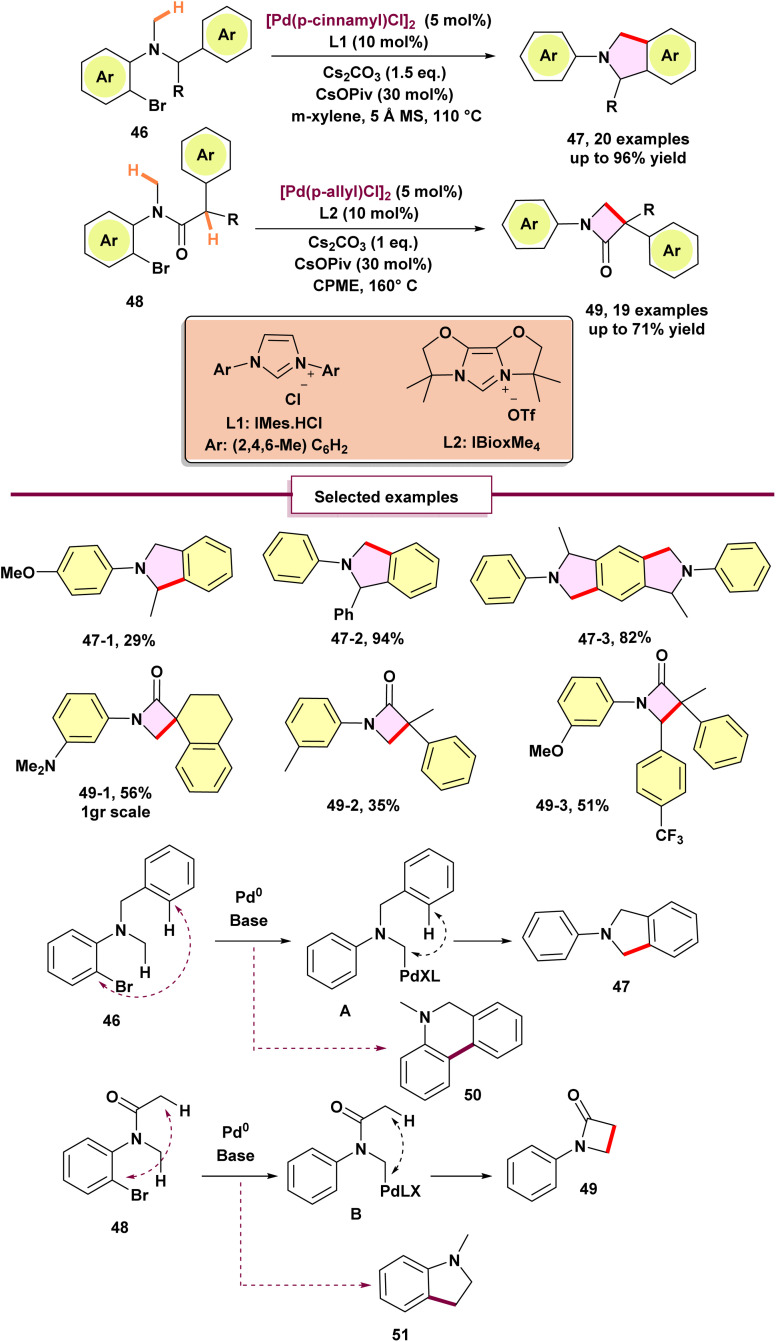
Synthesis of isoindolines and β-lactams through 1,4-palladium migration.

In 2022, Baudoin again developed a 1,4-palladium shift strategy by adding a suitable *N*-substituent to the previous example (Scheme 12).^67^ This modification helped trap the temporary σ-alkylpalladium intermediate (A and B), enabling the synthesis of isoindolines 47 and β-lactams 49. The preferred migration pathway competed with direct C–H arylation, resulting in byproducts like 5,6-dihydrophenanthridines 50 and oxindoles 51. Also, *N*-demethylation could happen without an electron-withdrawing group near the nitrogen atom. Nevertheless, it became clear that the 1,4-palladium shift pathway is significantly influenced by selecting the right ligand and base. For the isoindoline adduct, IMes HCl L1 was determined to be the most effective ligand for transforming the primary derivative substrate into the desired indoline 47-2 with a 94% NMR yield while minimizing the production of 5,6-dihydrophenanthridines 50 as a side product from direct C(sp^2^) arylation. Substrates with electron-withdrawing or electron-donating groups on the distal aryl ring were compatible, yielding the isoindolines products. Moreover, substrates containing electron-withdrawing groups in the *meta* or *para* positions of the *N*-aryl ring also reacted with high efficiency. Nonetheless, a substantial decrease in yield was noted when an electron-donating group was appended to the *N*-aryl ring 47-1. Ultimately, the employment of a symmetrical dibrominated substrate enabled a double reaction sequence including four times C–H activation, leading to the formation of hexahydro pyrroloisoindole 47-3 with an 82% yield. In the synthesis of the β-lactam, a stoichiometric amount of Cs_2_CO_3_ proved to be the optimal basic system for this transformation. Also, using stronger bases like LiHMDS or NaO*t*-Bu led to the selective formation of the oxindole, likely *via* an enolate arylation mechanism. IBiox-type ligands L2, particularly the Spiro cyclic IBioMe_4_, were found to yield the β-lactam product.

**Scheme 13:**
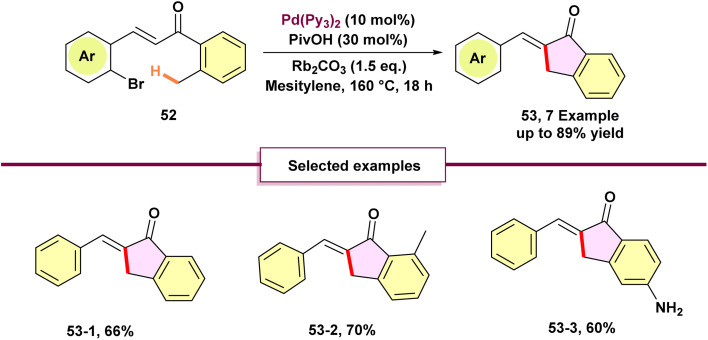
Synthesis of γ-lactams and indanones through 1,4-palladium migration.

Baudoin and colleagues in 2019 described a palladium^0^-catalyzed domino reaction that involves a 1,4-palladium shift coupled with C(sp^3^)–H activation for the synthesis of γ-lactams and indanones (Scheme 13).^68^ The reaction tolerates a range of *N*-alkyl amide substituents (ethyl, *tert*-butyl, cyclopropyl) and various aryl substituents, showing pronounced site-selectivity at the isopropyl α-C–H positions, avoids β-lactam byproducts, and sometimes favors C(sp^2^)–H activation over C(sp^3^)–H, enabling access to bicyclic γ-lactams (including 5,6-fused systems and enantiopure *N*-Boc piperazine derivatives) across 40–98% yields, with potential relevance to natural product–like frameworks such as pyrrolizidine and Stemona alkaloids. Furthermore, the methodology was employed in the formal synthesis of (–)-pyrrolam. The reaction sequence involved oxidative addition, followed by a 1,4-palladium migration to activate a C(sp^2^)–H bond, subsequent C(sp^3^)–H activation, and finally, formation of the product with palladium departure.

**Scheme 14:**
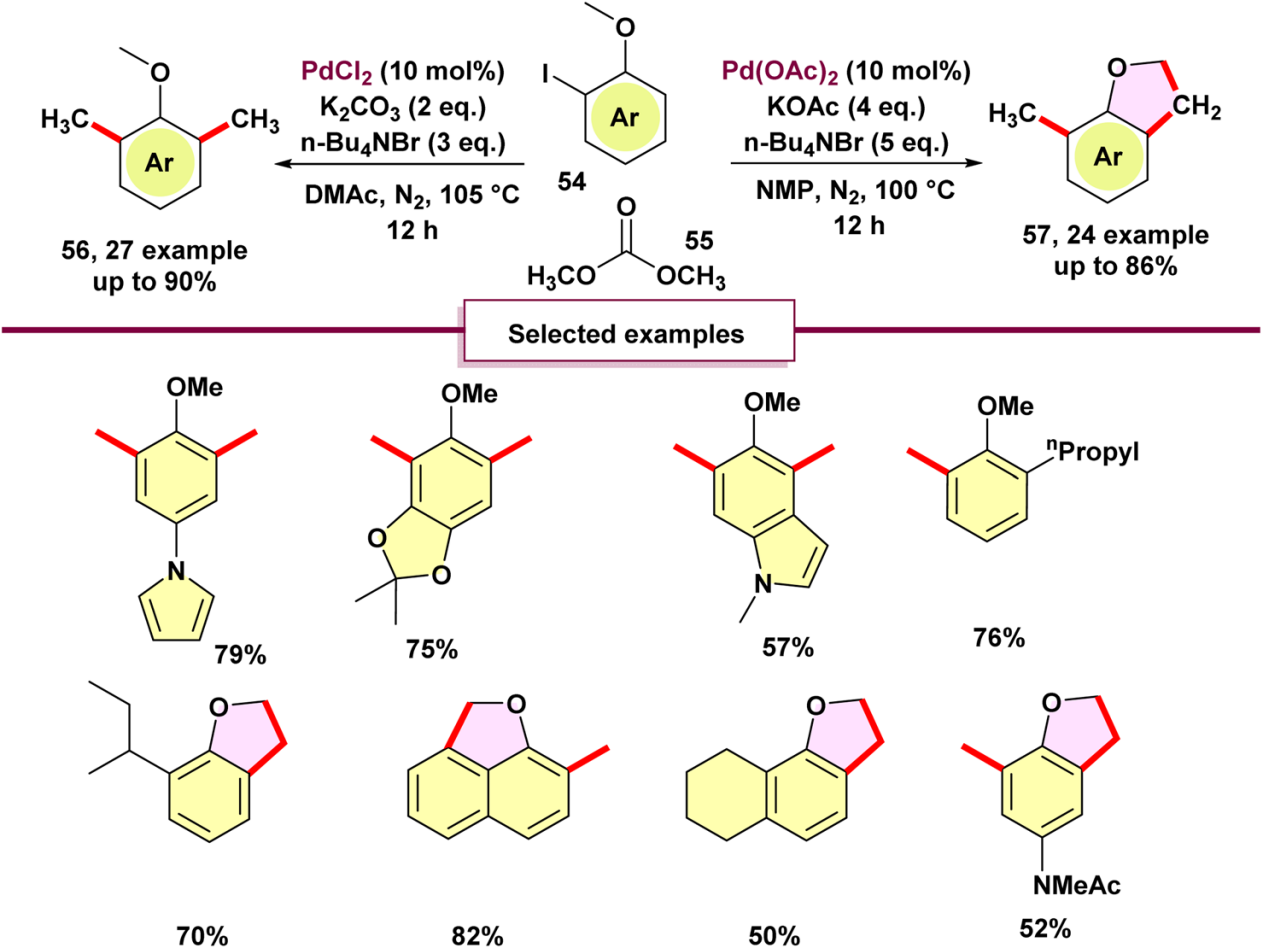
Palladium-catalyzed ipso, *meta*-dimethylation of *ortho*-substituted iodoarenes with dimethyl carbonate as the methyl source.

**Scheme 15:**
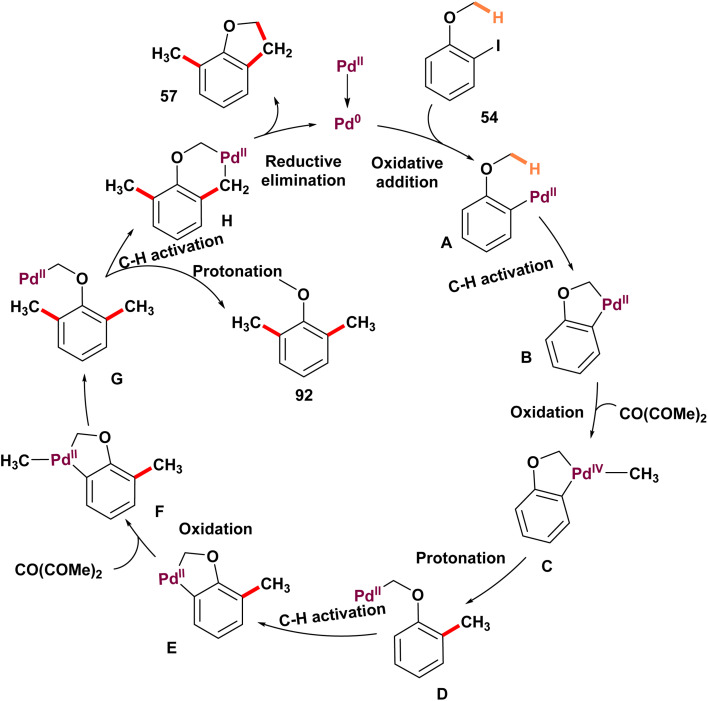
Mechanism of palladium-catalyzed ipso, *meta*-dimethylation of *ortho*-substituted iodoarenes with dimethyl carbonate as the methyl source.

**Scheme 16:**
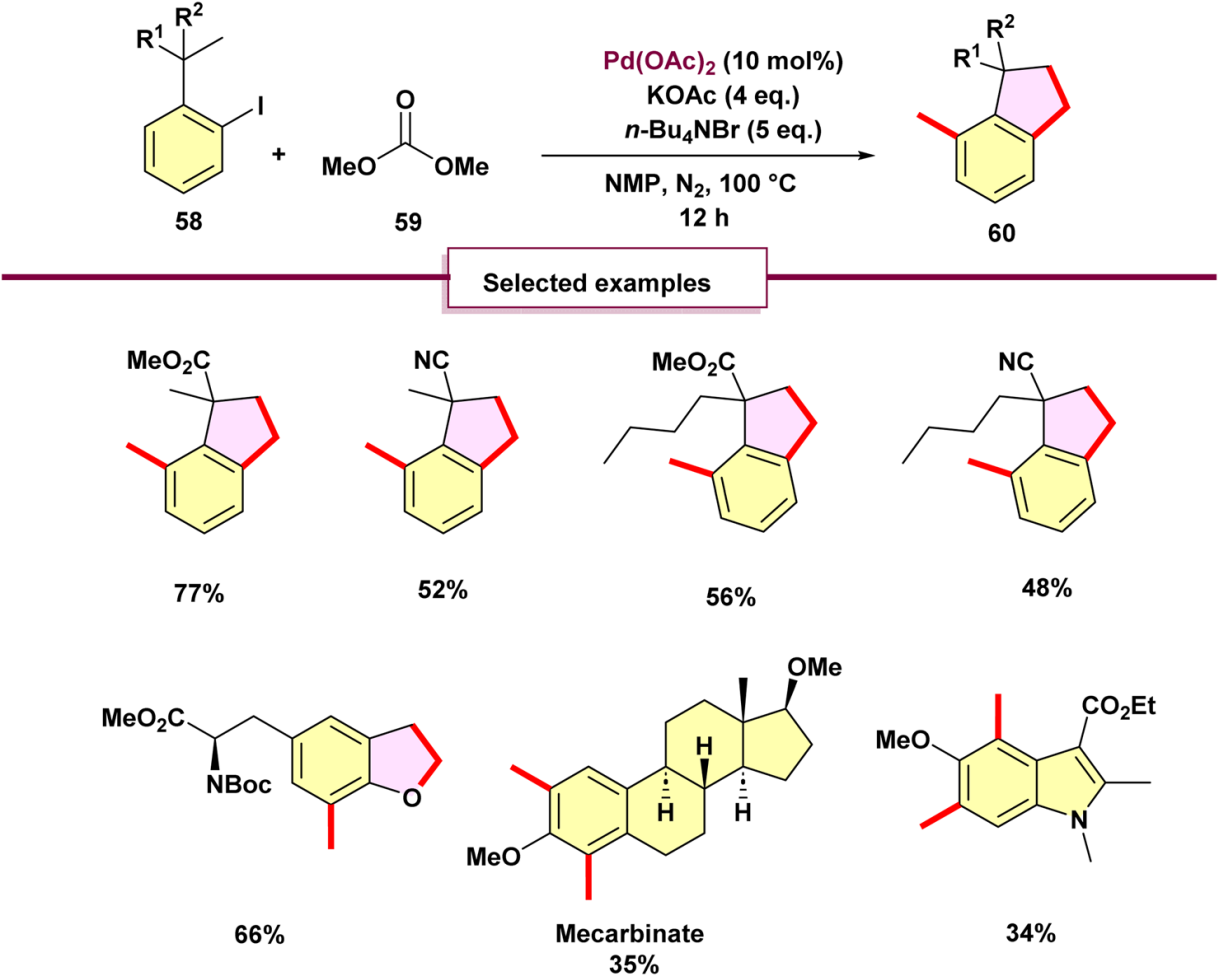
Synthesis of indanes with dimethyl carbonate as the methyl source.

In 2020, Zhang's team introduced a novel method for palladium-catalyzed C–H methylation of *ortho*-substituted iodoarenes 54, utilizing dimethyl carbonate (DMC) as the source of methyl groups (Scheme 14).^69^ This reaction marks the first use of DMC as a methylating agent in transition-metal-catalyzed cross-coupling reactions. In this innovative process, the iodo group serves as a transient directing group, enabling the methylation of the *meta*-C–H bond. Attempts with diethyl carbonate as a methyl source were unsuccessful, as no diethylated product was observed. Notably, product 57 emerged when Pd(OAc)_2_ was employed. The formation of 2,3-dihydrobenzofuran is interesting, requiring three C–H activation steps and three C–C bond formations. Initially, palladacycle B is produced *via* C(sp^3^)–H activation. Intermediate B then goes through oxidative addition with methyl halides that come from dimethyl carbonate (DMC), producing intermediate C. Subsequent reductive elimination of C yields intermediate D, which facilitates the cleavage of the aryl C–H bond to make a second palladacycle E. Intermediate E follows a similar sequence as B, introducing a second methyl group to generate F. In the presence of K_2_CO_3_, intermediate F is protonated by dimethylacetamide or methanol (formed from DMC), leading to the formation of the dimethylated product H. Significantly, DMC acts not only as the methyl source but also produces methanol, which aids in reducing palladium(ii) species. When KOAc is used, a third activation of methyl C–H bonds forms, resulting in the formation of palladacycle G. The reductive elimination of H produces product 56 and regenerates palladium(0) species, thus completing the catalytic cycle. Surprisingly, *ortho*-alkyl-substituted iodobenzenes 58 undergo dimethylation followed by cyclization to yield *ortho*-methylindanes 60 (Scheme 15). This method offers a novel route to synthesize substituted indanes, common in drugs and natural products.^70,71^ Preliminary mechanistic investigations were carried out, leading to a proposed pathway (Scheme 16).

**Scheme 17:**
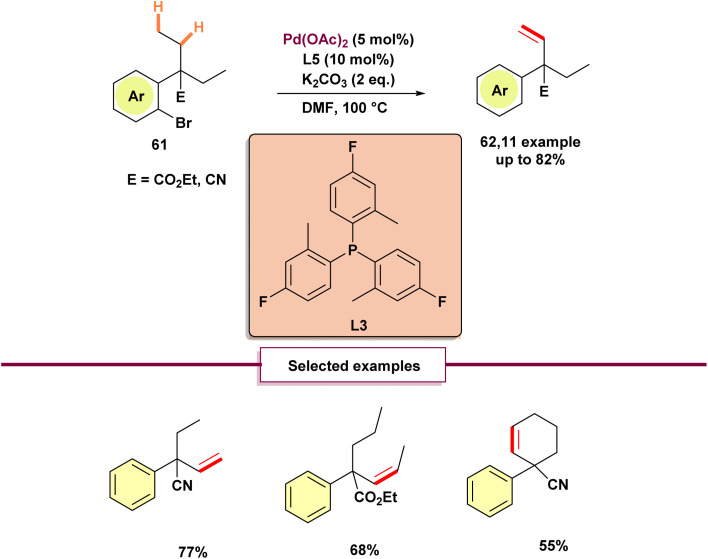
Palladium(0)-catalyzed C–H functionalization of benzylic alkyl groups.

Furthermore, in 2007, Baudoin and colleagues reported one of the first known examples of C(sp^3^)–H activation of compound 61 under significantly improved conditions (Scheme 17).^72^ While their initial study in 2003 required harsh reaction conditions (150 °C, 10 mol% palladium),^73^ this work emphasized the critical role of ligands, enabling the reaction to proceed in a milder environment. To demonstrate the practical utility of their approach, the authors successfully utilized their method in the synthesis of Verapamil, a widely used medication for managing high blood pressure.

**Scheme 18:**
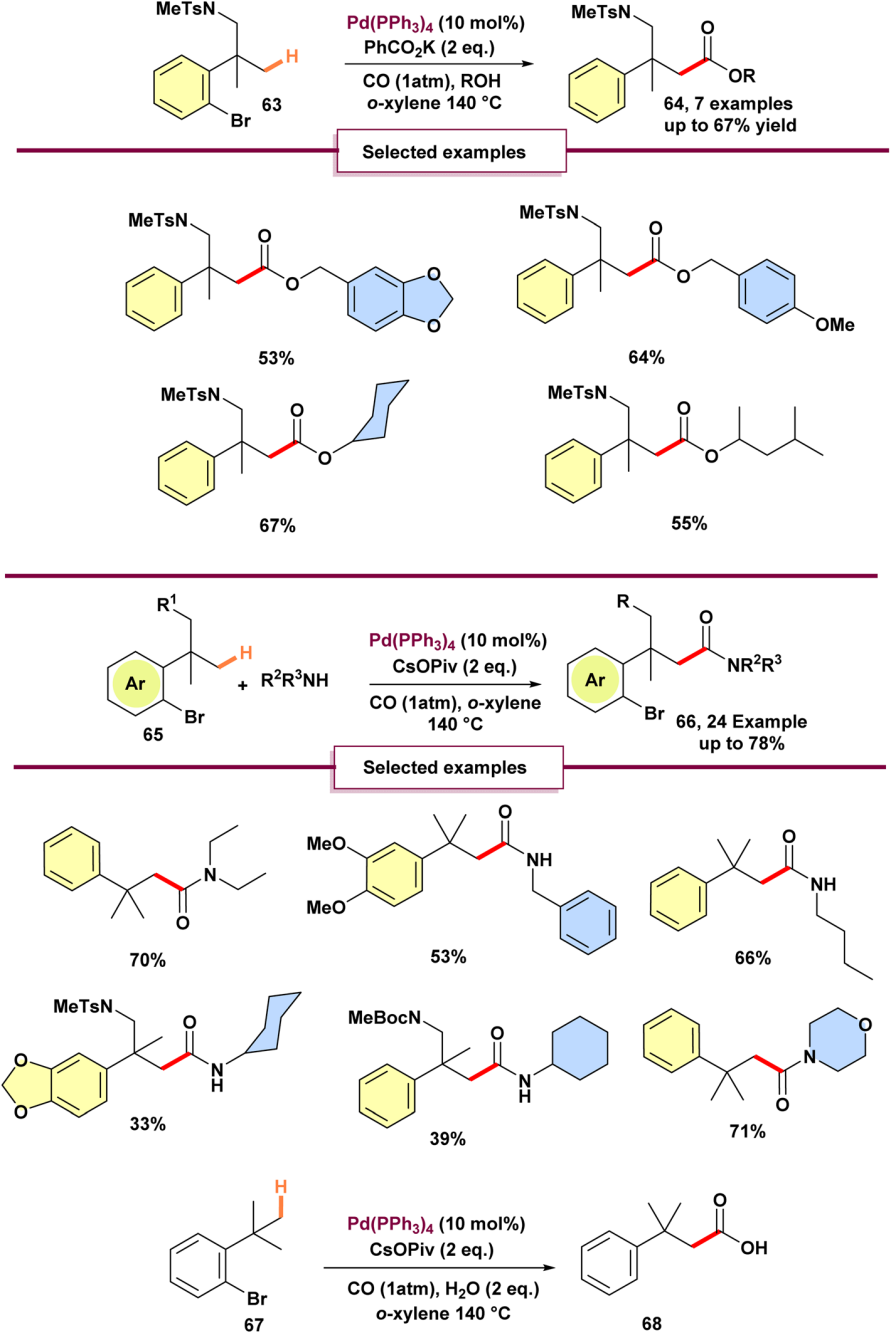
Synthesis of amides and esters by palladium(0)-catalyzed carbonylative C(sp^3^)–H activation.

In 2020, Baudoin reported the first carbonylative C(sp^3^)–H functionalization reaction involving a 1,4-palladium shift (Scheme 18).^74^ This method enabled the synthesis of various amides 66 and esters 64 featuring a quaternary β-carbon by amino- or alkoxycarbonylation. Mechanistic observation demonstrated that aminocarbonylation of the secondary alkylpalladium intermediate, formed *via* palladium migration, occurs rapidly when PPh_3_ is used as a ligand. This pathway preferentially results in amide formation, unlike prior reported indanone products.^75^ Presence of CO and an amine likely modifies the catalyst through ligand exchange, thereby influencing the reaction's energy barrier. Notably, in the absence of an amine, using PPh_3_ as the ligand resulted in carboxylic acid 68 as the major product. Further optimized reaction condition was required to achieve efficient ester synthesis *via* alkoxy carbonylation. Potassium benzoate was identified as a superior base for this transformation. Extending the reaction duration to 27 hours was essential to improve the yield, likely due to the lower nucleophilicity of alcohols relative to amines.^75^

**Scheme 19:**
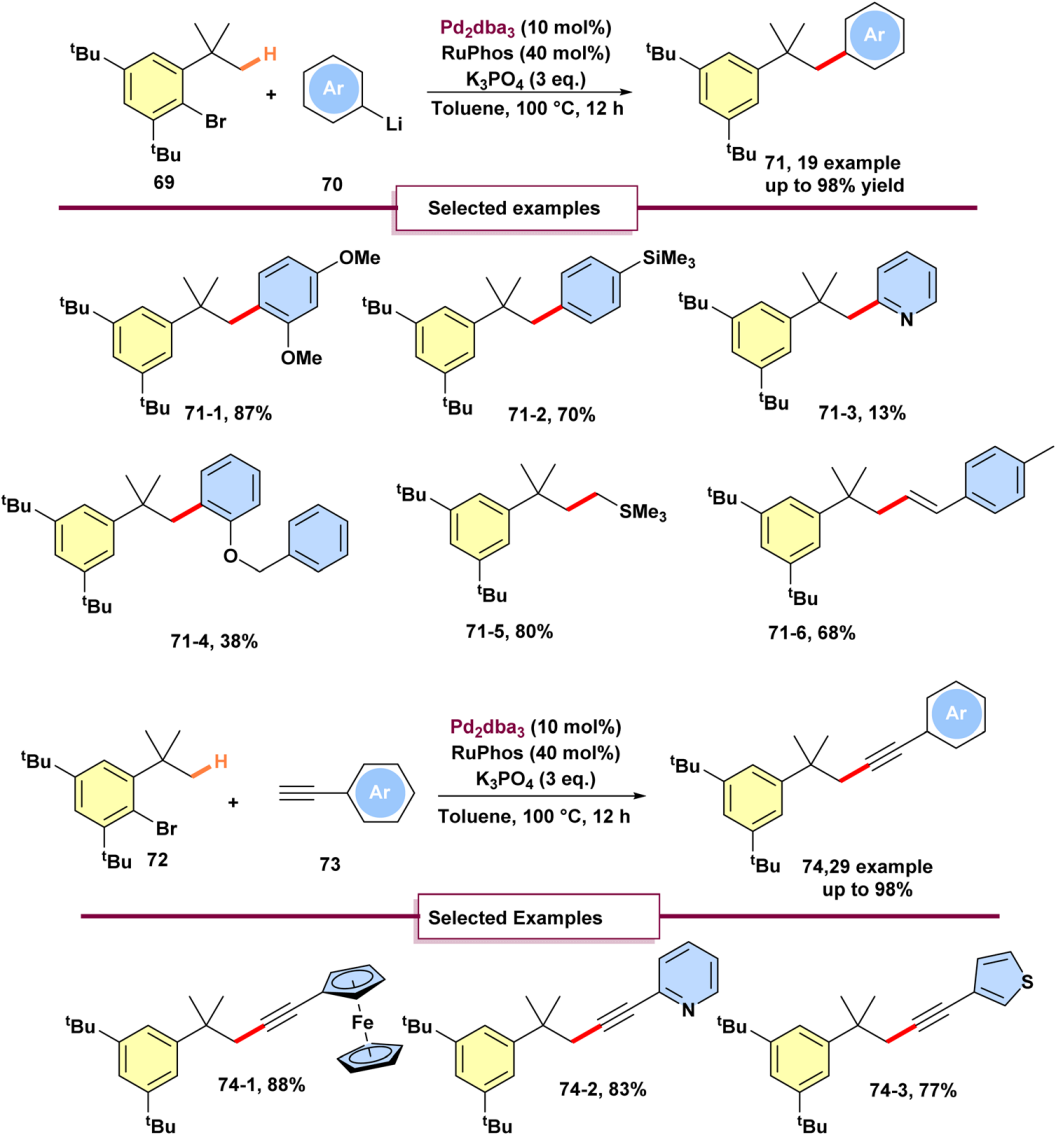
Palladium-catalyzed intermolecular arylation and alkynylation with organolithiums and terminal alkynes.

In 2022, Wong and Shui Peng, inspired by Buchwald's work in 2011 on the intermolecular arylation of inert C(sp^3^)–H bonds using boronic acids.^76^ They demonstrated intermolecular arylation, alkenylation, alkylation, and alkynylation of unactivated C(sp^3^)–H bonds employing organolithium compounds 70 and terminal alkynes 73, facilitated by a 1,4-palladium shift mechanism (Scheme 19).^77^ Notably, they introduced the first catalytic protocol enabling alkynylation between C(sp)–H and C(sp^3^)–H bonds. A range of aryllithium reagents 36 was evaluated, focusing on variations in substituent groups. Notably, the use of *ortho-*, *meta*-, and *para*-anisyllithiums resulted in consistently good to excellent yields. In contrast, a significant reduction in yield was observed when 2-pyridinyllithium (71-3) was employed. To assess the scope of the reaction, alkenyllithium and alkyllithium reagents were examined. The experimental findings revealed that both alkenyllithium and (trimethylsilylmethyl) lithium reacted efficiently with Buchwald's substrate, leading to C(sp^3^)–C(sp^2^) and C(sp^3^)–C(sp^3^) bond formation 71-5, 71-6. These results highlight the feasibility of utilizing alkenyllithium and alkyllithium reagents in palladium-catalyzed remote functionalization of C(sp^3^)–H bonds. Furthermore, encouraged by the promising results from these cross-coupling reactions, the investigation was extended to develop C(sp^3^)–C(sp) cross-coupling involving terminal alkynes 73 and unactivated C(sp^3^)–H bonds. Terminal alkynes 73, with electron-donating and withdrawing substituted at the *ortho*-, *meta*-, and *para*-positions and different heterocyclic groups, underwent coupling effectively to yield the expected products 74. To show the practical applicability of their methodology, they conducted the reaction on a larger scale, approximately 1.0 gram. The scale-up experiment successfully yielded the corresponding product with a satisfactory yield. Furthermore, in 2025, Feng and coworkers performed an aryl-to-alkyl 1,4-palladium migration coupled with a Sonogashira reaction to deliver migratory products in moderate to good yields.^78^ The reaction was conducted with Feng, avoiding the need for strict hindrance groups on the aryl reagent, and facilitated the synthesis of diverse products.

**Scheme 20:**
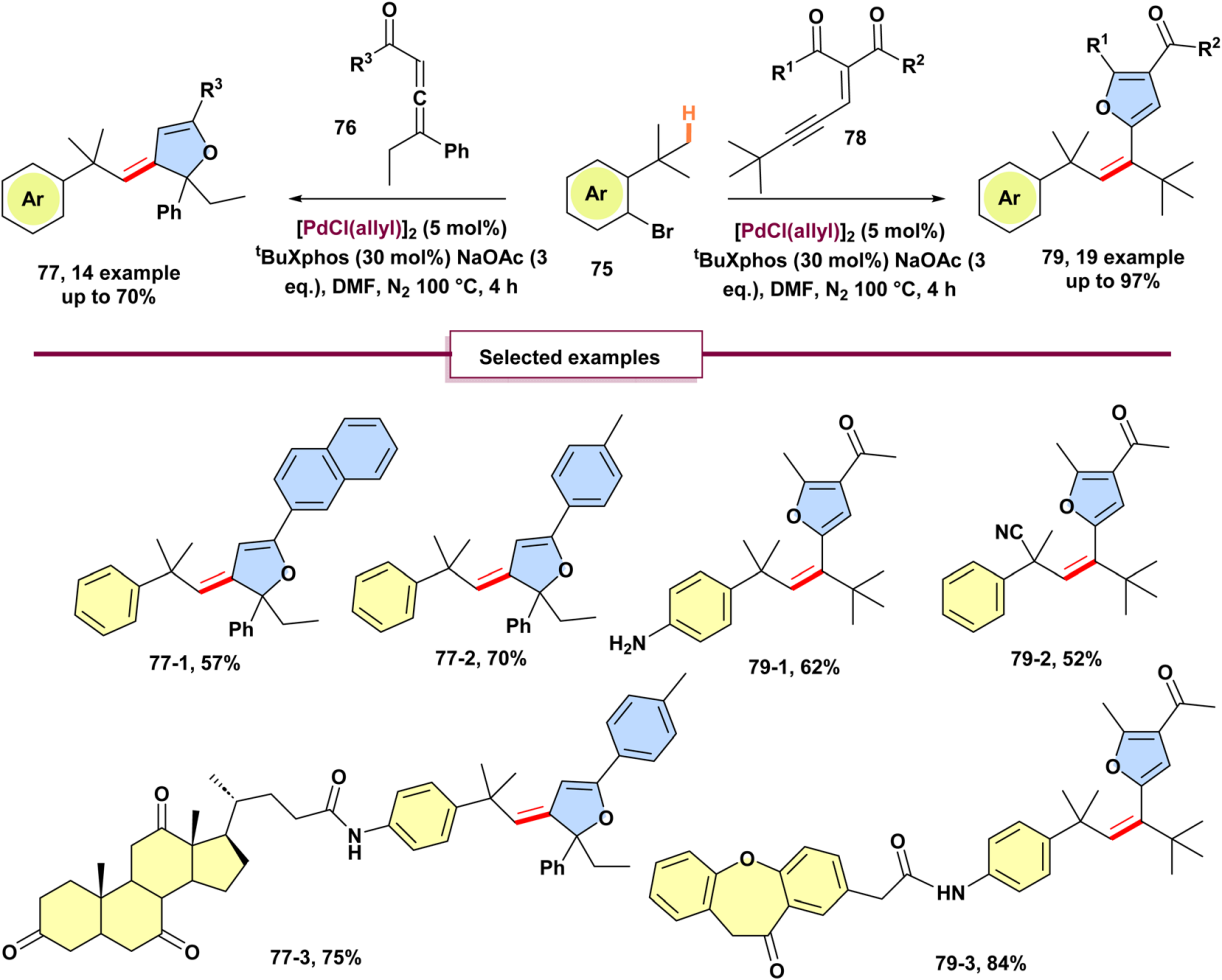
Palladium-catalyzed unactivated C(sp^3^)–H olefination mediated by donor/donor carbenes.

**Scheme 21:**
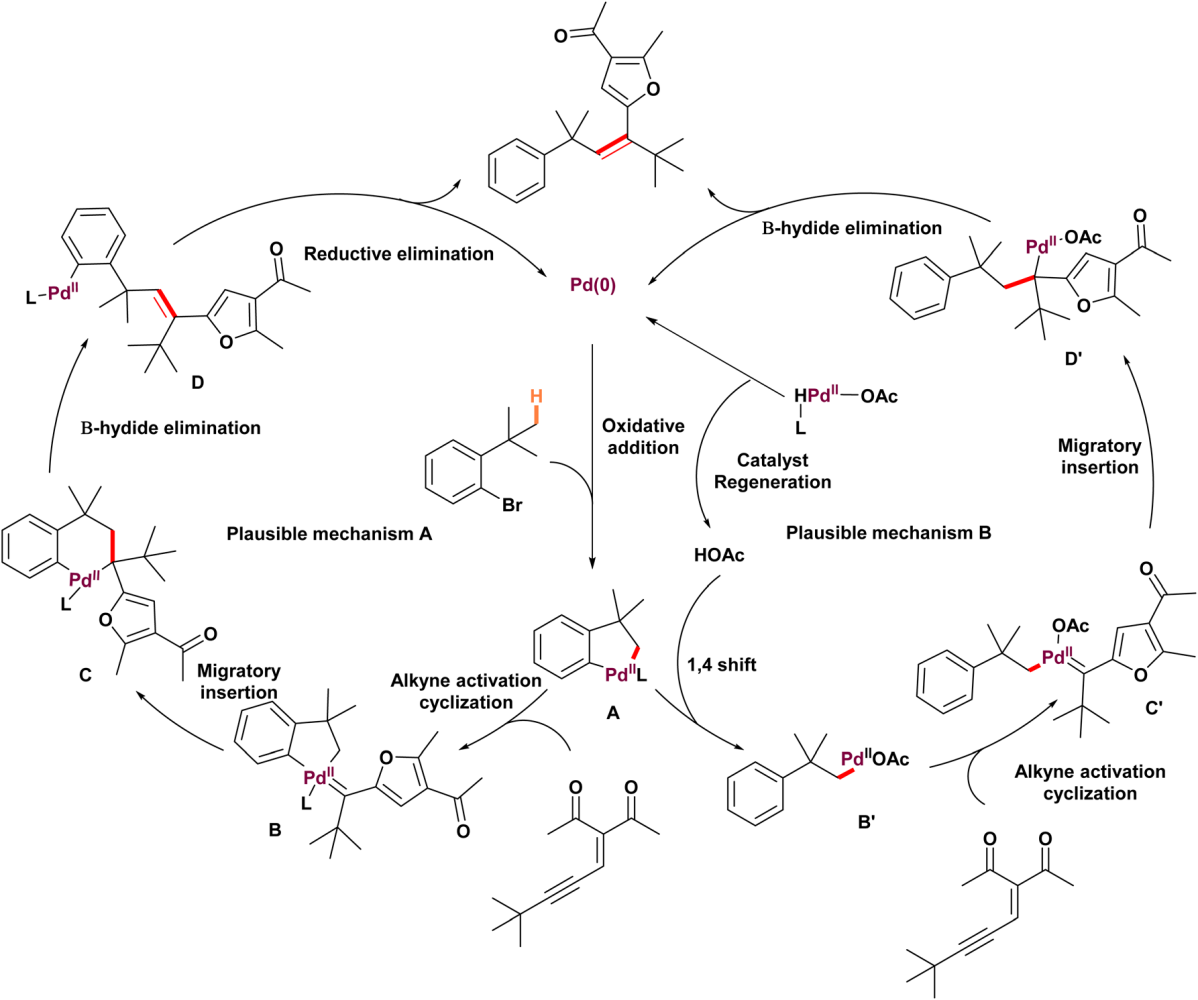
Mechanism of palladium-catalyzed unactivated C(sp^3^)–H olefination mediated by donor/donor carbenes.

In 2019, Yang and colleagues established a procedure for intermolecular palladium-catalyzed olefination of inactivated Csp^3^–H bonds through palladium migration involving initial C (sp^3^)–H bond activation followed by formation of carbene and migratory insertion (Scheme 20).^68^ This was accomplished using ene-yne-ketones 78 and allenyl ketones 76 as donor/donor carbene precursors. In addition to standard derivatization, it is significant that alkenes substituted with furans and dihydrofurans were synthesized in moderate (52 to 84% yield) yields from modified compounds like repaglinide, isoxepac, mycophenolic acid, adapalene, and dehydrocholic acid. For example, isoxepac 79-3, a non-steroidal anti-inflammatory drug with analgesic effects, was effectively transformed into a furan derivative. The method accommodates tertiary alkyl substrates bearing cyano and ester groups, while aryl, secondary, and primary alkyl substituents show limited reactivity, likely due to steric hindrance or β-H elimination; substrates with free amines on the aryl bromide furnish in good yield without N–H insertion side products. The authors also investigated the reaction mechanism and proposed two plausible catalytic cycles (Scheme 21). To distinguish the preferred pathway, DFT calculations were performed. Beginning from the key palladacycle intermediate A, two mechanistic routes were considered. In Path A, alkyne activation and cyclization follow the migratory insertion step (Cycle A). In contrast, Path B proceeds *via* protonation before alkyne activation and cyclization. Path A was found to involve a high-energy transition state (B to C) during migratory insertion, likely due to the unfavorable cleavage of two strong palladium–C bonds within the five-membered palladacycle intermediate B. The computational studies ultimately supported Path B as the energetically favored pathway.

### 4.2. C–N coupling by 1,4 palladium migration

**Scheme 22:**
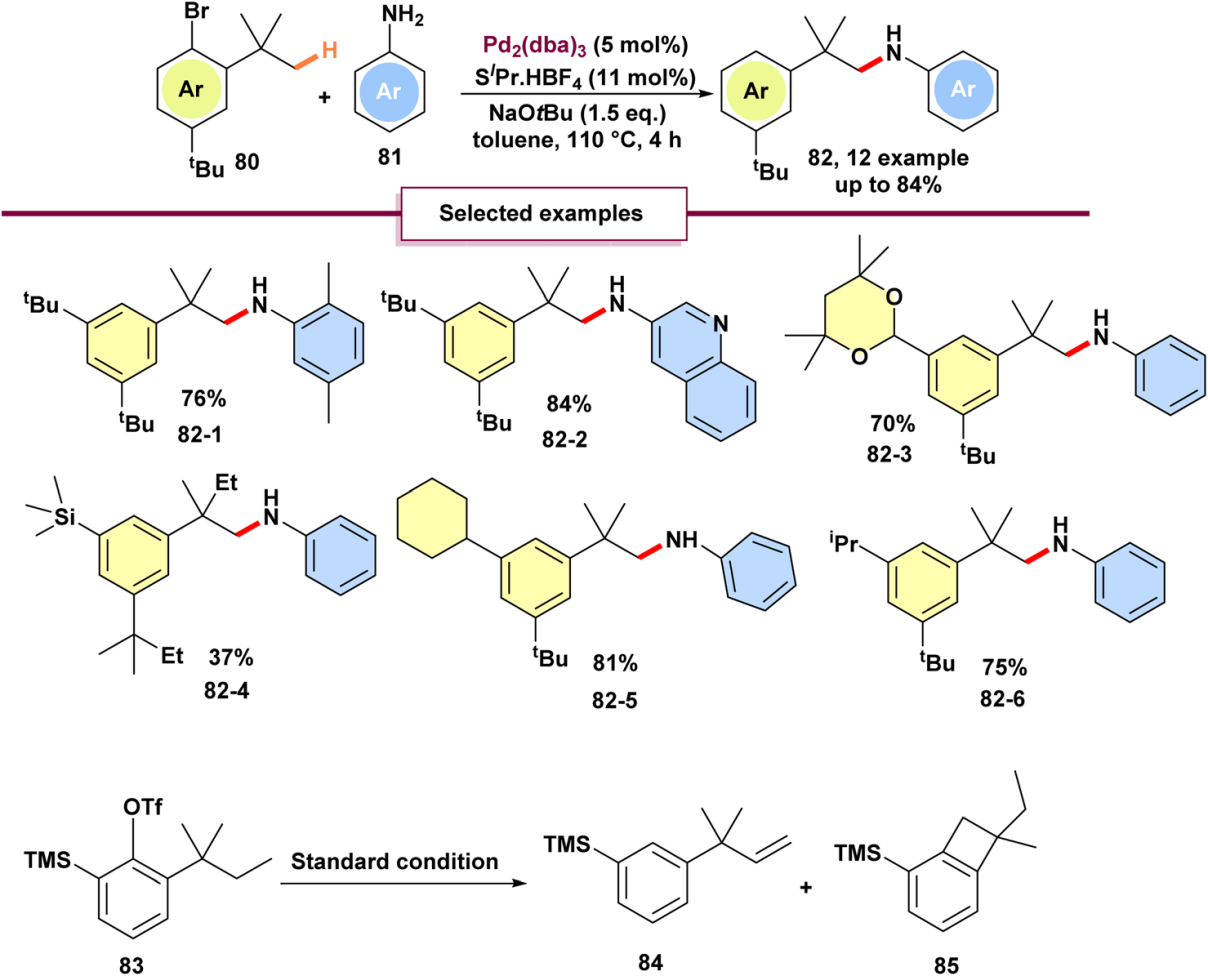
Palladium(0)-catalyzed intermolecular amination of unactivated C(sp^3^)–H bonds.

Buchwald and his team introduced an approach for the amination of homobenzylic C(sp^3^)–H bonds using 1-bromo-2,4,6-tri-*tert*-butylbenzene 80, which involves a 1,4-palladium migration process (Scheme 22).^79^ Both electron-deficient and electron-rich anilines successfully yielded the expected products in high yields, including anilines with *ortho*-alkyl substituents. However, the authors noted that *N*-substituted anilines and alkyl amines were not compatible with the current procedure. Additionally, less sterically hindered substrates resulted exclusively in diaryl amine products, suggesting that the *ortho*-methyl group lacks sufficient steric bulk to prevent direct C–N cross-coupling. No C–H amination was observed for isopropyl, cyclopentyl, or cyclohexyl groups, indicating a high selectivity for the methyl groups of *tert*-butyl moieties. Similarly, no C–H amination occurred with TMS groups. Under identical conditions, compound 83 failed to undergo C–H amination, instead yielding a mixture of olefin 84 and benzocyclobutene 85.

### 4.3. C–P coupling by 1,4 palladium migration

**Scheme 23:**
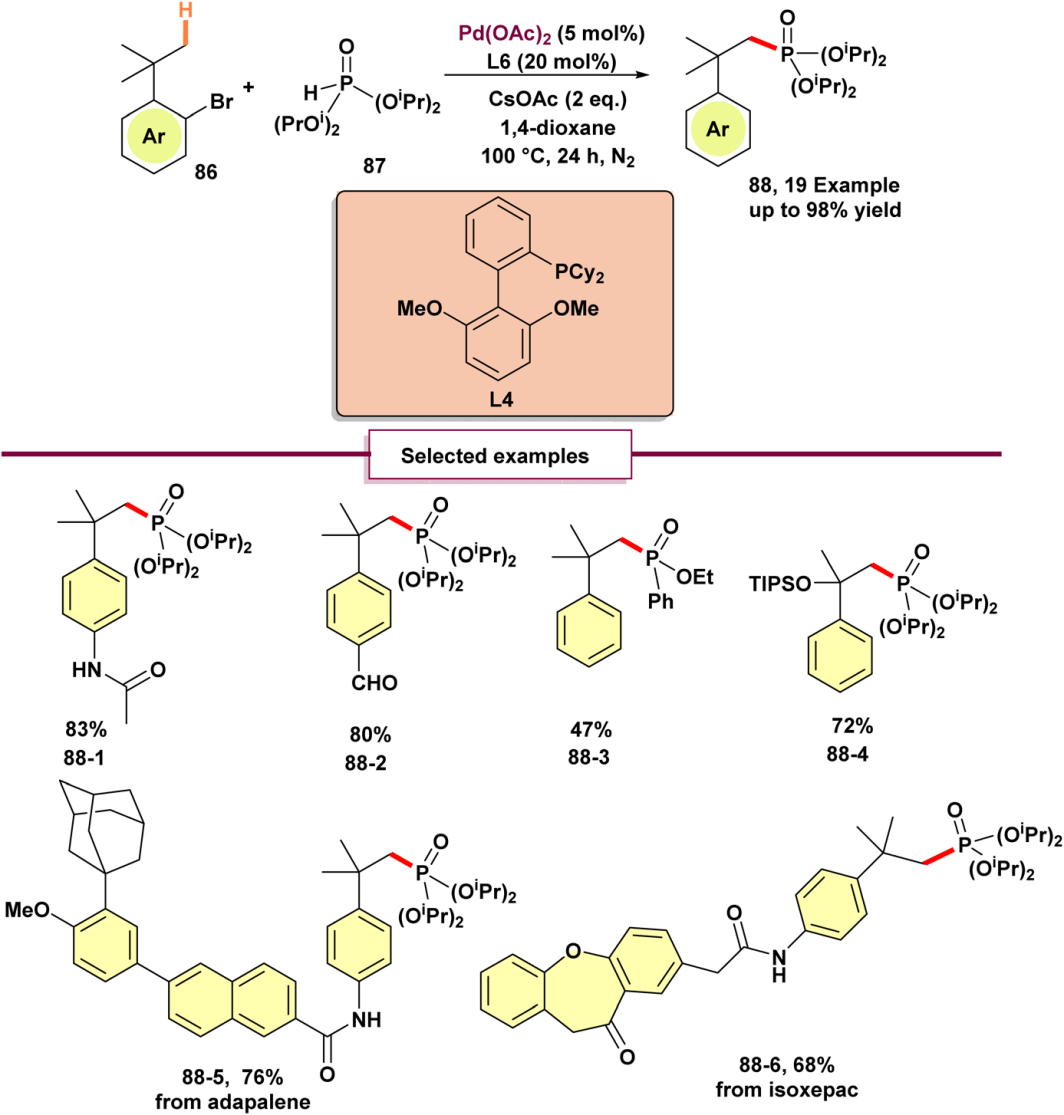
Phosphorylation of C(sp^3^)–H bonds *via* 1,4-palladium migration.

In contrast to other heteroatom coupling partners, phosphorus reagents pose a challenge due to their strong coordination ability, which can deactivate the catalyst and preferentially interact with the palladium(ii) species initially formed. Consequently, a different route—C–H phosphorylation achieved through 1,4-palladium migration—had not been reported prior to 2022, when Feng and Fu showcased efficient phosphorylation of alkyl C(sp^3^)–H bonds by employing an appropriate ligand (L4) in conjunction with a 1,4-palladium migration strategy (Scheme 23).^80^ The reaction is highly ligand-dependent, with most optimization efforts focused on ligand selection. The presence of CsOAc is essential because the acetate ion participates in the C–H activation step *via* a CMD mechanism. The reaction showed broad compatibility with various benzene rings bearing electron-donating and electron-deficient functional groups, affording the corresponding products 88 in good to excellent yields. Notably, high efficiency was also observed when a methyl group on the aryl bromide substrate was substituted with an ethyl group, a TIPS-protected alcohol, or a sulfonamide. This protocol was utilized on aryl bromide substrates from drug compounds like adapalene and isoxepac as well (88-5, 88-6), highlighting the possibility of application of this phosphorylation technique in medicinal chemistry.

### 4.4. C–O coupling by 1,4 palladium migration

**Scheme 24:**
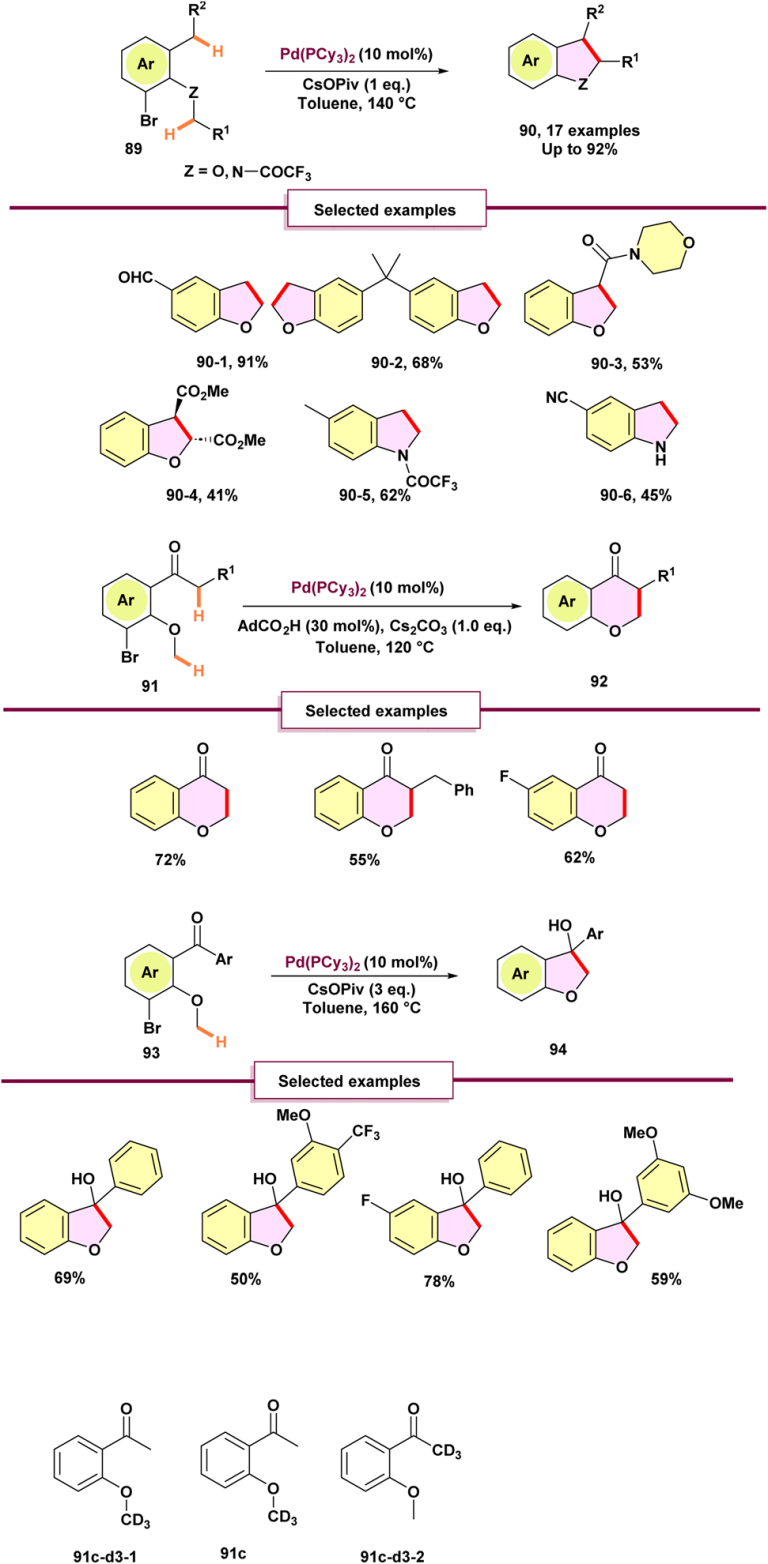
Redox-neutral coupling between two C(sp^3^)–H bonds enabled by 1,4-palladium shift for the synthesis of fused heterocycles.

The formation of a Transition-metal-catalyzed C–C bond through double C–H bond cleavage, known as cross-dehydrogenative coupling, has become an important transformation in organic synthesis. However, the formation of C(sp^3^)–C(sp^3^) bonds using this method is still relatively underexplored compared to C(sp^2^)–C(sp^2^) and C(sp^2^)–C(sp^3^) bond formation. In 2019, Baudoin and collaborators introduced a palladium migration-based method for C(sp^3^)–C(sp^3^) bond formation (Scheme 24).^81^ Following a brief optimization, the authors discovered that using the Pd(PCy_3_)_2_ complex along with Cs(Opiv) as a base led to the successful formation of 2,3-dihydrobenzofuran 90 with an excellent yield. This study marks the first instance of a 1,4-palladium shift next to a nitrogen atom, promoting C–C bond formation. While the reaction is currently restricted to primary and acidic secondary alkyl C–H bonds, it offers a complementary method for synthesizing indoline 90-6 through straight palladium^0^-catalyzed C(sp^3^)–H arylation. The authors aimed to expand this methodology to the formation of six-membered rings 92. This effort was successful, as the reaction was expanded to arylalkyl ketones 91, tolerating aromatic ring substituents and other alkyl groups. Notably, the resulting chroman-4-ones are invaluable heterocycles with significant potential in drug discovery.^82–86^ In the absence of an enol resonance form, the organopalladium intermediate resulting from the 1,4-palladium shift can attack the ketone, leading to the formation of a tertiary alcohol. Under more stringent conditions, arylketones underwent a 1,4-palladium shift followed by nucleophilic addition, yielding the dihydrobenzofuran scaffold product 94 in moderate yields.

The deuterated substrate 91c-d3-1 does not convert to chromanones under standard conditions, suggesting a very large kinetic isotope effect and turnover-limiting initial C(sp3)–H activation, while equimolar 9c/9c-d3-2 yields a chromanone with 50% deuterium at the acarbonyl, indicating no primary KIE at that position and supporting rate limitation by the first C(sp^3^)–H activation.

### 4.5. C–Si coupling by 1,4 palladium migration

**Scheme 25:**
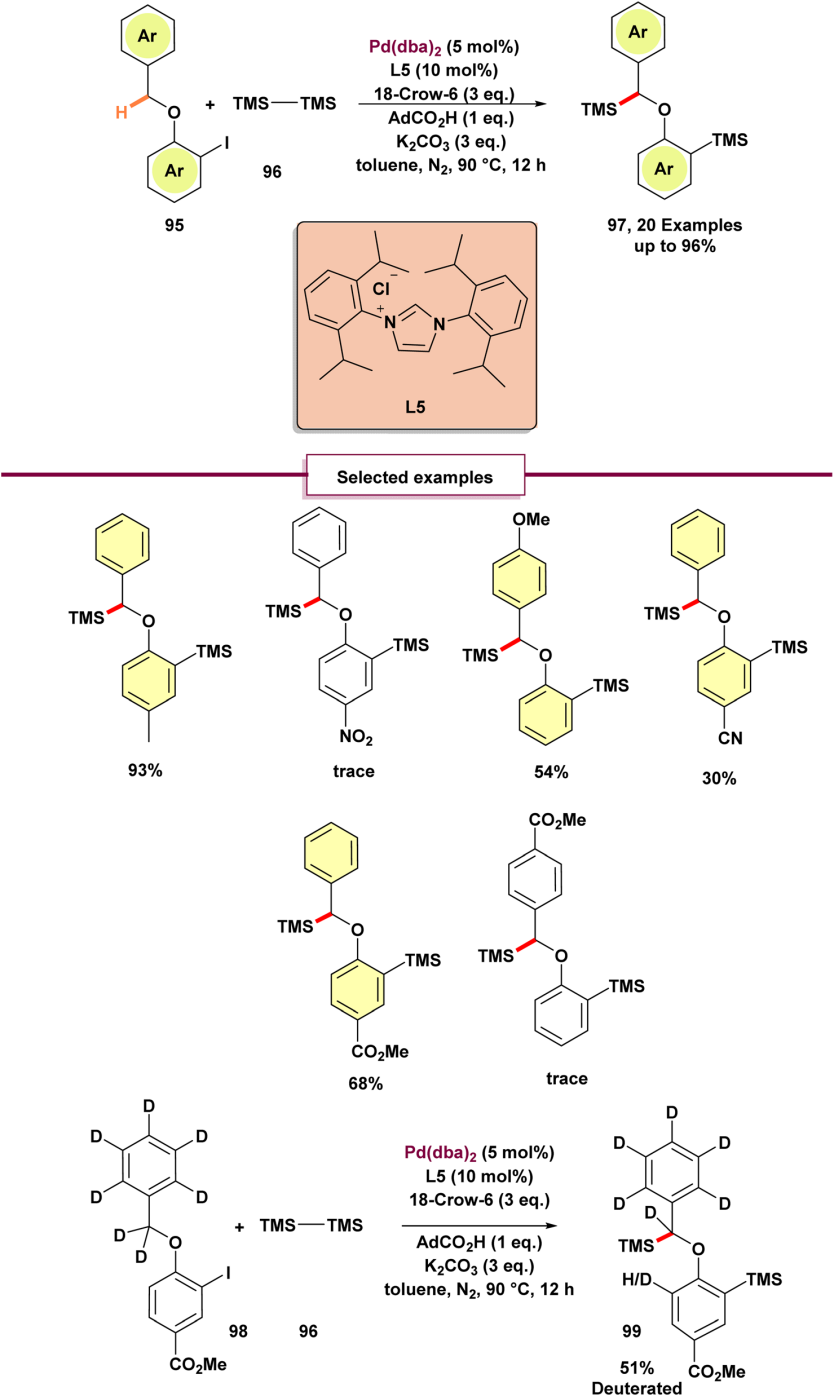
Palladium(0)-catalyzed intermolecular methylene C(sp^3^)–H silylation by using N-heterocyclic carbene ligands.

**Scheme 26:**
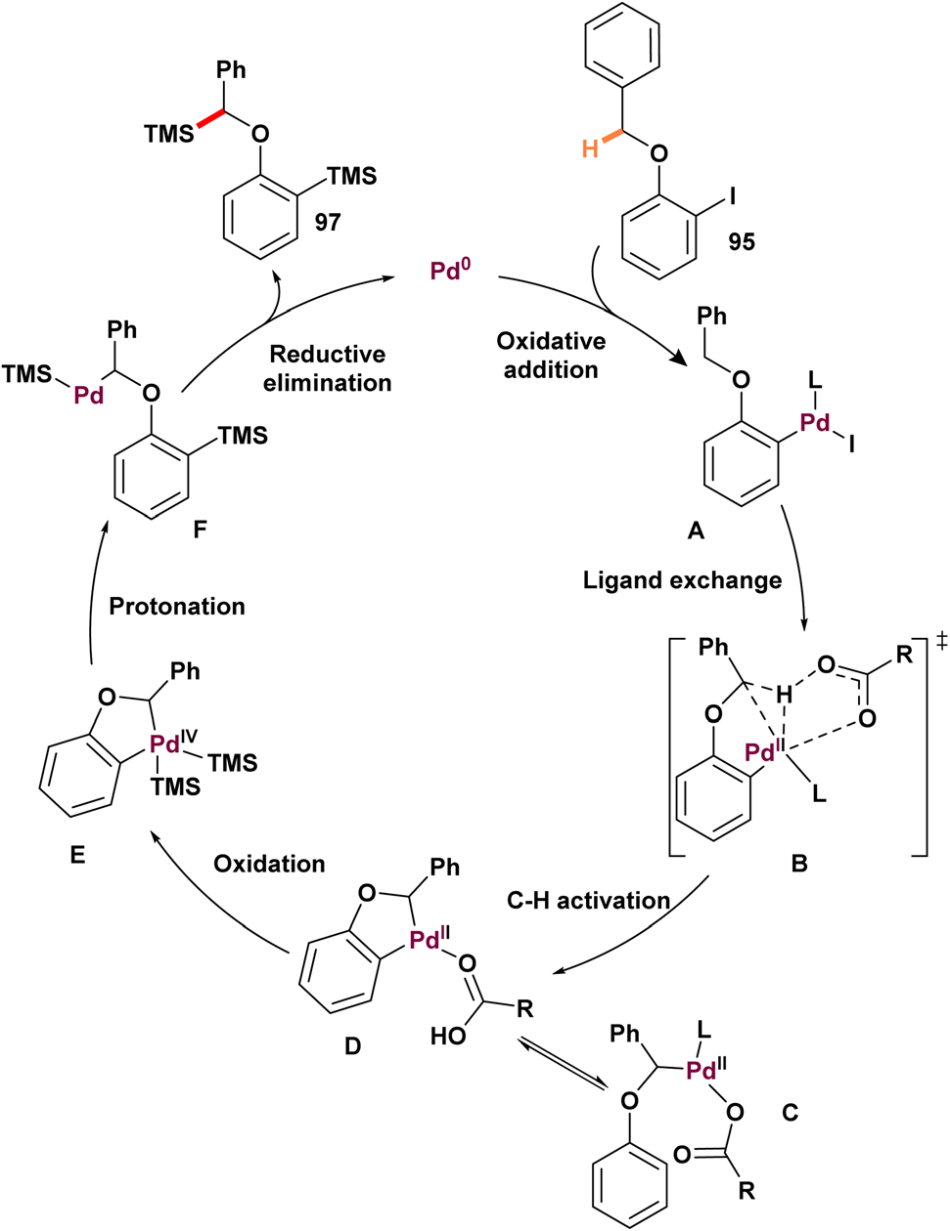
Proposed mechanism for palladium(0)-catalyzed intermolecular methylene C(sp^3^)–H silylation by using N-heterocyclic carbene ligands.

In 2024, Zhang and Wang described the earliest example of a palladium (0)-catalyzed intermolecular methylene C(sp^3^)–H functionalization reaction (Scheme 25).^87^ This groundbreaking work demonstrated the disilylation of a variety of 1-(benzyloxy)-2-iodobenzenes 95 through the selective silylation of methylene C(sp^3^)–H bonds. Under optimized conditions, substrates with electron-donating or electron-withdrawing groups on the iodobenzene ring afforded moderate to excellent yields, except for nitro-substituted derivatives, which were unable to produce the desired product. Additionally, the presence of an ester group on the tolyl moiety suppressed the reaction, leading to the formation of only the monosilylated product. Subjecting the deuterated derivative 98 to the standard conditions yielded the disilylated product. Notably, partial deuteration was observed at the *ortho*-carbon of product 99, suggesting migration of the benzylic deuterium to the *ortho* position. Additionally, the kinetic isotope effect (KIE) was measured, revealing a value of 2.8, which supports the conclusion that the rate-determining step is C–H activation. Based on the research results and previous studies, the proposed catalytic cycle begins with the oxidative addition of 96 (benzyloxy)-2-iodobenzene to palladium (0), forming intermediate A. Phenyl-palladium(ii) species facilitates activation of the benzylic C–H bond, forming palladacycle C. This C–H activation is proposed to take place through an intramolecular base-mediated concerted metalation-deprotonation mechanism (intermediate B). The phenyl–palladium bond in intermediate C can be protonated by the benzylic hydrogen, generating complex D. Complex D may then undergo aryl C–H activation, reverting to intermediate C. Subsequently, Carbon reacts with hexamethyl disilane through oxidative addition, producing palladium(iv) species E. Finally, intermediate E undergoes dual reductive elimination, yielding the expected product 97 and regenerating the palladium (0) catalyst (Scheme 26).

## 5. Conclusions

In recent years, 1,4-palladium migration has become an increasingly important tool in the field of C(sp^3^)–H functionalization. Since it was first discovered in 1992, this process has been refined and widely applied to a variety of organic transformations. Essentially, it allows palladium to move from one carbon atom to a distant one, separated by two atoms, often forming a stable five-membered palladacycle. This feature helps activate difficult-to-reach C–H bonds located far from the directing group. Researchers have successfully used this migration to functionalize a range of positions, including vinylic, aryl, alkyl, benzylic, and homobenzylic sites, producing complex molecules such as aryl, acyl, and imidoyl derivatives. This technique has played a crucial role in constructing complex natural products and pharmaceutical compounds with high precision. The typical process involves palladium first inserting into a C–H or C–X bond through oxidative addition, then migrating to a remote site, followed by further functionalization steps like reductive elimination. Scientists have improved these reactions by optimizing ligands, bases, and electrophiles, broadening their scope and improving selectivity. Recent studies have demonstrated exciting applications in drug modification, cyclizing molecules to form fused rings, and creating heterocyclic compounds. Although challenges like side reactions and reversible migration still exist, ongoing research is expanding the versatility of this approach. In many examples, the 1,4-palladium migration employs traceless directing groups, suggesting that these directing groups may play a unique or special role in the 1,4-palladium migration process. Additionally, for C(sp^3^)–H functionalization facilitated by 1,4-palladium migration, ligands were sometimes not used, or were limited to ligands with an or two l-type dentate, while ligands containing an X-type dentate did not seem to be employed. For the same reaction class, bases were observed in almost all examples, underscoring the essential role of the base in these processes. Overall, 1,4-palladium migration is now recognized as a powerful and versatile method for activating remote C–H bonds, revolutionizing modern organic synthesis and offering new opportunities in drug discovery and material science.

## Conflicts of interest

There are no conflicts to declare.

## Data Availability

No primary research results, software or code have been included and no new data were generated or analysed as part of this review.
